# Dexmedetomidine pretreatment alleviates ropivacaine-induced neurotoxicity via the miR-10b-5p/BDNF axis

**DOI:** 10.1186/s12871-022-01810-6

**Published:** 2022-09-26

**Authors:** Weicai Xu, Xiaojun Li, Long Chen, Xiaopan Luo, Sheliang Shen, Jing Wang

**Affiliations:** 1 Rehabilitation Medicine Center, Department of Anesthesiology, Zhejiang Provincial People’s Hospital, Affiliated People’s Hospital, Hangzhou Medical College, Hangzhou, China; 2 Department of General Practice, The Second Affiliated Hospital of Zhejiang University School of Medicine, Hangzhou, China

**Keywords:** Dexmedetomidine, Ropivacaine, miR-10b-5p, BDNF, Neurotoxicity

## Abstract

**Background:**

Ropivacaine is commonly applied for local anesthesia and may cause neurotoxicity. Dexmedetomidine (DEX) exhibits neuroprotective effects on multiple neurological disorders. This study investigated the mechanism of DEX pretreatment in ropivacaine-induced neurotoxicity.

**Methods:**

Mouse hippocampal neuronal cells (HT22) and human neuroblastoma cells (SH-SY5Y) were treated with 0.5 mM, 1 mM, 2.5 mM, and 5 mM ropivacaine. Then the cells were pretreated with different concentrations of DEX (0.01 μM, 0.1 μM, 1 μM, 10 μM, and 100 μM) before ropivacaine treatment. Proliferative activity of cells, lactate dehydrogenase (LDH) release, and apoptosis rate were measured using CCK-8 assay, LDH detection kit, and flow cytometry, respectively. miR-10b-5p and BDNF expressions were determined using RT-qPCR or Western blot. The binding of miR-10b-5p and BDNF was validated using dual-luciferase assay. Functional rescue experiments were conducted to verify the role of miR-10b-5p and BDNF in the protective mechanism of DEX on ropivacaine-induced neurotoxicity.

**Results:**

Treatment of HT22 or SH-SY5Y cells with ropivacaine led to the increased miR-10b-5p expression (about 1.7 times), decreased BDNF expression (about 2.2 times), reduced cell viability (about 2.5 times), elevated intracellular LDH level (about 2.0–2.5 times), and enhanced apoptosis rate (about 3.0–4.0 times). DEX pretreatment relieved ropivacaine-induced neurotoxicity, as evidenced by enhanced cell viability (about 1.7–2.0 times), reduced LDH release (about 1.7–1.8 times), and suppressed apoptosis rate (about 1.8–1.9 times). DEX pretreatment repressed miR-10b-5p expression (about 2.5 times). miR-10b-5p targeted BDNF. miR-10b-5p overexpression or BDNF silencing reversed the protective effect of DEX pretreatment on ropivacaine-induced neurotoxicity, manifested as reduced cell viability (about 1.3–1.6 times), increased intracellular LDH level (about 1.4–1.7 times), and elevated apoptosis rate (about 1.4–1.6 times).

**Conclusions:**

DEX pretreatment elevated BDNF expression by reducing miR-10b-5p expression, thereby alleviating ropivacaine-induced neurotoxicity.

**Supplementary Information:**

The online version contains supplementary material available at 10.1186/s12871-022-01810-6.

## Background

Ropivacaine, a long-acting amide-linked local anesthetic, has presented extensive application in clinical anesthesia and pain management [1]. Relative to other local anesthetics, ropivacaine is featured by a better separation of sensory and motor nerve blocking, fewer systemic reactions, and lower cardiotoxicity [2]. Nevertheless, emerging studies have also provided supportive evidence that exposure to ropivacaine in vivo and in vitro can lead to considerable neurotoxicities [3, 4]. Intrathecal injection of ropivacaine is demonstrated as an underlying triggering factor of neuronal injury in rats, resulting in tissue edema as well as morphological alternation and degeneration of neurons [5]. Ropivacaine is also reported to exacerbate rat pheochromocytoma PC12 cell injury and apoptosis in a concentration-dependent manner [6]. Since ropivacaine-induced nerve injury has already occurred at clinical concentrations [7], the prevention and management of ropivacaine-induced neurotoxicity has become an urgent issue in current research.

Dexmedetomidine (DEX) is a selective a2-adrenoceptor agonist that bears unique pharmacodynamic properties conducive to sedation and anesthesia in perioperative medicine [8]. DEX has drawn extensive attention due to its multi-organ protection advantages, especially in the fields of neuroprotection [9]. DEX is frequently used as an adjuvant to local anesthetics, with proven efficacy in prolonging the duration of peripheral nerve block [10]. The combination of ropivacaine and DEX is the current trend, and DEX may confer protective effects against ropivacaine-induced neuronal injury [11]. The addition of DEX to ropivacaine notably alleviates ropivacaine-induced neurotoxicity by repressing sciatic nerve cell apoptosis in rats [12]. DEX also protects PC12 cells from ropivacaine injury by facilitating the proliferation and suppressing apoptosis of PC12 cells [13]. Clarifying the neuroprotective mechanisms of DEX in ropivacaine-induced neurotoxicity can translate into prominent clinical benefits.

microRNAs (miRNAs), a class of small non-coding RNAs with 20–25 nucleotides in length, modulate gene expression post-transcriptionally via binding to the 3' untranslated region (UTR) of their messenger RNAs (mRNAs) [14]. Recently, the crucial implications of miRNAs in local anesthetic-induced neurotoxicity have been identified, suggesting that miRNAs may be novel targets in neurotoxicity prevention [15, 16]. Moreover, differentially expressed miRNAs are also implicated in the neuroprotective mechanisms of DEX [17]. As a member of the miRNA family, miR-10b-5p is reported to be associated with cognitive dysfunction by impairing hippocampal neurogenesis [18]. Knockdown of miR-10b-5p attenuates neuronal apoptosis, alleviates pathological injury, and reduces inflammation responses in rats with Alzheimer's disease (AD) [19]. Importantly, DEX can attenuate neurological injury in ischemic stroke rats and enhance the viability of neurons via inhibition of miR-10b-5p expression [20]. Brain-derived neurotrophic factor (BDNF) is one of the most extensively distributed neurotrophins in the central nervous system, which acts as an instructive mediator of functional and structural plasticity in the central nervous system, influencing the adult neurogenesis in the hippocampus [21]. Thus, manipulating BDNF pathways represents a viable treatment approach to a variety of neurological and psychiatric disorders [22]. Upregulation of BDNF has been demonstrated to inhibit neuronal apoptosis and alleviate neurotoxicity induced by methamphetamine [23]. Accordingly, we speculated whether DEX pretreatment can upregulate BDNF expression through miR-10b-5p to regulate neuronal viability and protect against ropivacaine-induced neurotoxicity. In the present study, we treated HT22 and SH-SY5Y cells with ropivacaine to determine the possible protective mechanism of DEX pretreatment in ropivacaine-induced neurotoxicity, hoping to find therapeutic targets for ropivacaine-induced neurotoxicity in clinical anesthesia surgery.

## Materials and methods

### Cell culture

Mouse hippocampal neuronal cell line (HT22) was obtained from Millipore (Billerica, MA, USA) and human neuroblastoma cell line (SH-SY5Y) was supplied by American Type Culture Collection (Manassas, Virginia, USA). All cells were cultured in Dulbecco’s modified Eagle’s medium (DMEM) (Gibco, Grand Island, NY, USA) containing 10% fetal bovine serum, 100 IU/mL penicillin G sodium, and 100 mg/mL streptomycin sulfate at 37℃ with 5%CO_2_.

### Cell treatment

For ropivacaine-induced neurotoxicity, the experimental cells were added with 0.5 mM, 1 mM, 2.5 mM, and 5 mM ropivacaine in the medium. The medium was not changed until the end of the relevant experiments. The control cells were treated with dimethyl sulphoxide. The cells were subjected to cell counting kit-8 (CCK-8) assay for the detection of cell viability. To determine the protective effect of DEX, the medium was supplemented with 0.01 μM, 0.1 μM, 1 μM, 10 μM, and 100 μM DEX for 2 h of pre-treatment, and then, 2.5 mM ropivacaine was added to the medium, followed by CCK-8 assay.

### Cell transfection

24 h before ropivacaine treatment, HT22 or SH-SY5Y cells were transfected with miR-10b-5p-mimic, small interfering (si)-BDNF-1, si-BDNF-2, and their negative controls (NCs) using Lipofectamine 3000 (Invitrogen Inc., Carlsbad, CA, USA). Human and mouse miR-10b-5p-mimic, si-BDNF-1, si-BDNF-2, and their negative controls were designed and synthesized by GenePharma (Shanghai, China).

### CCK-8 assay

The transfected HT22 cells or SH-SY5Y cells were seeded into 96-well plates (5 × 10^3^ cells/well) and then treated with ropivacaine or DEX as mentioned above. After 24, 48, and 72 h of incubation, the cells in each well were treated with 10 μL CCK-8 solution and cultured at 37℃ for 2 h. The absorbance at 450 nm was measured using a microplate reader (Biotek).

### Lactate dehydrogenase (LDH) detection

HT22 cells or SH-SY5Y cells were seeded into 96-well plates and treated as mentioned above. Then, the cells were collected and lysed with cell lysis buffer (Beyotime, Shanghai, China). The supernatant was collected after 10 min of centrifugation. LDH release in the supernatant was detected using LDH cytotoxicity kit (ab197004, Abcam Inc., Cambridge, MA, USA) to determine the damage degree of HT22 cells or SH-SY5Y cells.

### Apoptosis detection

Cell apoptosis was measured using Annexin V fluorescin isothiocyanate (FITC)/propidium iodide (PI) apoptosis detection kit and subjected to flow cytometry analysis. Briefly, the treated cells were collected, rinsed with cold phosphate buffered saline (PBS), re-suspended in PBS, and stained with 10 μL Annexin V/FITC in the dark for 30 min. Finally, the filtered cells were placed in PBS and analyzed using a flow cytometer (BD Biosciences, San Jose, CA, USA).

### Reverse transcription quantitative polymerase chain reaction (RT-qPCR)

The total RNA was extracted using TRIzol reagent (Invitrogen) and reverse transcribed into cDNA using TaqMan miRNA reverse transcription kit (Applied Biosystems, Inc., Carlsbad, CA, USA). RT-qPCR was performed using Brilliant II Fast SYBR green qPCR master mix (Agilent Technologies) and MyiQ Real-time PCR system. Primer sequences are shown in Table 1. The relative expression of genes was calculated using the 2^−△△Ct^ method, with GAPDH and U6 as the internal reference [19]. In each statistical analysis, the first group was set as "1" as the control.

**Table 1:** qPCR primers

	**Forward Primer (**5′-3′**)**	**Reverse Primer (**5′-3′**)**
mmu-miR-10b-5p	GCCGAGTACCCTGTAGAA	CTCAACTGGTGTCGTGGA
mmu-BDNF	AGAAGAGTGATGACCATC	ATAAATCCACTATCTTCC
mmu-GAPDH	TTAAGAGGGATGCTGCCC	CAGGGTTTCTTACTCCTT
has -miR-10b-5p	GCCGAGTACCCTGTAGAA	CTCAACTGGTGTCGTGGA
has-BDNF	AGAAGAGTGATGACCATC	ATAAATCCACTATCTTCC
has-GAPDH	CTCAACTACATGGTTTAC	CCAGGGGTCTTACTCCTT

*Note: miR-10b-5p* microRNA-10b-5p, *BDNF* brain derived neurotrophic factor, *GAPDH* glyceraldehyde-3-phosphate dehydrogenase

### Western blot

The total protein was extracted using radio-immunoprecipitation assay lysis buffer (Invitrogen) and quantified using bicinchoninic acid assay kits (Beyotime). Then, 50 μg protein sample was separated by 10% SDS-PAGE and transferred onto polyvinylidene fluoride membranes. The membranes were blocked with 5% skim milk for 1 h and incubated with anti-BDNF (1:1000, ab108319, Abcam) and anti-GAPDH (1:2500, ab9485, Abcam) at 4℃ overnight. Afterward, the membranes were incubated with peroxidase-labeled goat anti-rabbit IgG and developed using an enhanced chemiluminescence reagent. The gray value was analyzed using Image J software version 1.8.0 (NIH, Bethesda, MD, USA), with GAPDH as the internal reference. In this experiment, blots were cut prior to hybridisation with antibodies, so the full-length blots could not be obtained, and all replicates performed were shown in the Supplementary Information File.

### Bioinformatics

The downstream targets of miR-10b-5p were predicted through the TargetScan (http://www.targetscan.org/vert_72/) [24] and miRDB (http://mirdb.org/) [25] databases. The binding site of miR-10b-5p and BDNF was predicted through the TargetScan database.

### Dual-luciferase reporter gene assay

The wild-type (WT) and mutant-type (MUT) sequences of BDNF 3’UTR containing miR-10b-5p binding site were cloned into pGL3 vector (Promega Corporation, Madison, WI, USA) to generate pGL3-BDNF 3’UTR-WT and pGL3-BDNF 3’UTR-MUT plasmids. The above plasmids were co-transfected with miR-10b-5p-mimic or mimic-NC into HT22 cells and SH-SY5Y cells using Lipofectamine 3000. The relative luciferase activity was measured using the dual-luciferase assay system (Promega).

### Statistical analysis

Data analysis and map plotting were performed using the SPSS 21.0 (IBM Corp., Armonk, NY, USA) and GraphPad Prism 8.0 (GraphPad Software Inc., San Diego, CA, USA). Measurement data are expressed as mean ± standard deviation. The *t* test was adopted for comparisons between the two groups. One-way or two-way analysis of variance (ANOVA) was employed for the comparisons among multiple groups, following Tukey's multiple comparison test. A value of *p* < 0.05 indicated a statistical difference.

## Results

### DEX pretreatment reduced ropivacaine-induced neurotoxicity

**Fig. 1:**
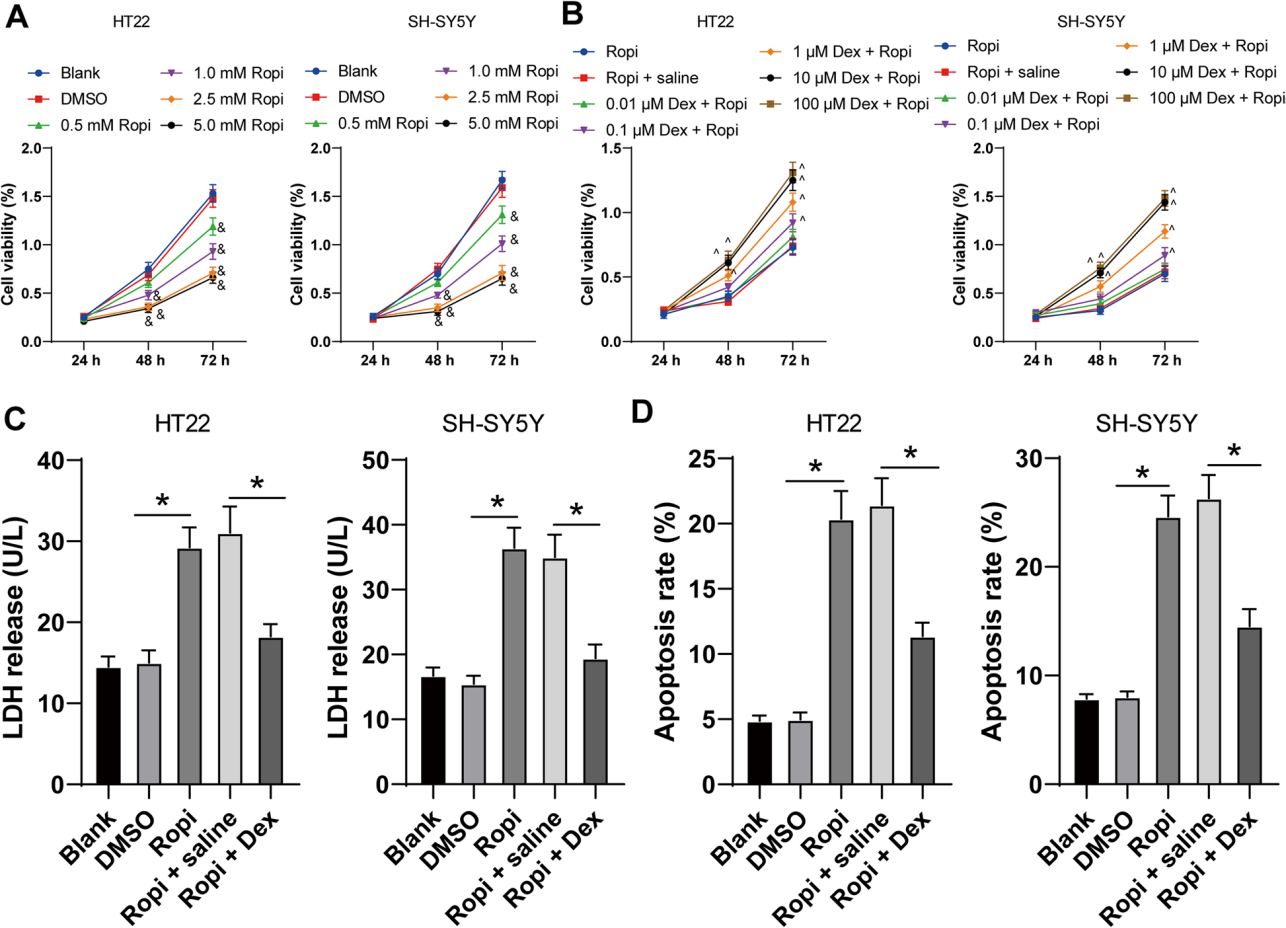
Dexmedetomidine pretreatment reduced ropivacaine-induced neurotoxicity. HT22 and SH-SY5Y cells were treated with different concentrations of ropivacaine (Ropi), with DMSO as negative control. **A** The cell viability was measured using CCK-8 assay. The cells were pretreated with different concentrations of dexmedetomidine (Dex) and then treated with 2.5 mM Ropi. **B** Cell viability was measured using CCK-8 assay, and the optimal concentration of Dex was determined as 10 μM. Then, 10 μM Dex-pretreated cells (Dex group) and 2.5 mM Ropi-treated cells (Ropi group) were subjected to LDH release detection using LDH detection kit (**C**) and apoptosis detection using flow cytometry (**D**). The cell experiment was repeated 3 times independently. Data are presented as mean ± standard deviation. Data in panels **A-B** were analyzed using two-way ANOVA, and data in panels **C-D** were analyzed using one-way ANOVA, followed by Tukey's multiple comparisons test, ^&^*p* < 0.05, compared with the Blank group, ^^^*p* < 0.05, compared with the Ropi group, ^*^p < 0.05

To investigate the protective effect of DEX on ropivacaine-induced neurotoxicity, we treated HT22 and SH-SY5Y cells with different concentrations of ropivacaine (0.5 mM, 1 mM, 2.5 mM, and 5 mM) and evaluated the cell viability using CCK-8 assay. The results revealed that ropivacaine treatment notably reduced the viability of HT22 cells and SH-SY5Y cells, and the cell viability was decreased with the increase of ropivacaine concentration (*p* < 0.05, Fig. 1A). When the concentration of ropivacaine was 2.5 mM, the cell viability reached the lowest, and there was no significant difference in the effect of ropivacaine on cell viability when the concentration increased to 5 mM (*p* > 0.05, Fig. 1A). Hence, we selected ropivacaine at the concentration of 2.5 mM for subsequent experiments. After ropivacaine treatment, HT22 and SH-SY5Y cells had increased LDH release (*p* < 0.05, Fig. 1C) and elevated apoptosis rate (*p* < 0.05, Fig. 1D, Supplementary Fig. 1). Then, the cells were pretreated with different concentrations of DEX (0.01 μM, 0.1 μM, 1 μM, 10 μM, and 100 μM). It was found that the viability of HT22 cells and SH-SY5Y cells pretreated with DEX was notably higher than that of cells not pretreated with DEX (*p* < 0.05, Fig. 1B). When the DEX concentration was 10 μM, the cell viability reached the highest, and when the DEX concentration reached 100 μM, there was no significant difference in the effect of DEX on cell viability (*p* > 0.05, Fig. 1B). Hence, we used 10 μM DEX for cell pretreatment and 2.5 mM ropivacaine to induce neurotoxicity. After DEX pretreatment, LDH release in HT22 and SH-SY5Y cells was decreased (*p* < 0.05, Fig. 1C) and apoptosis rate was declined (*p* < 0.05, Fig. 1D). All these indicated that DEX pretreatment reduced ropivacaine-induced neurotoxicity.

### DEX pretreatment suppressed miR-10b-5p expression

**Fig. 2:**
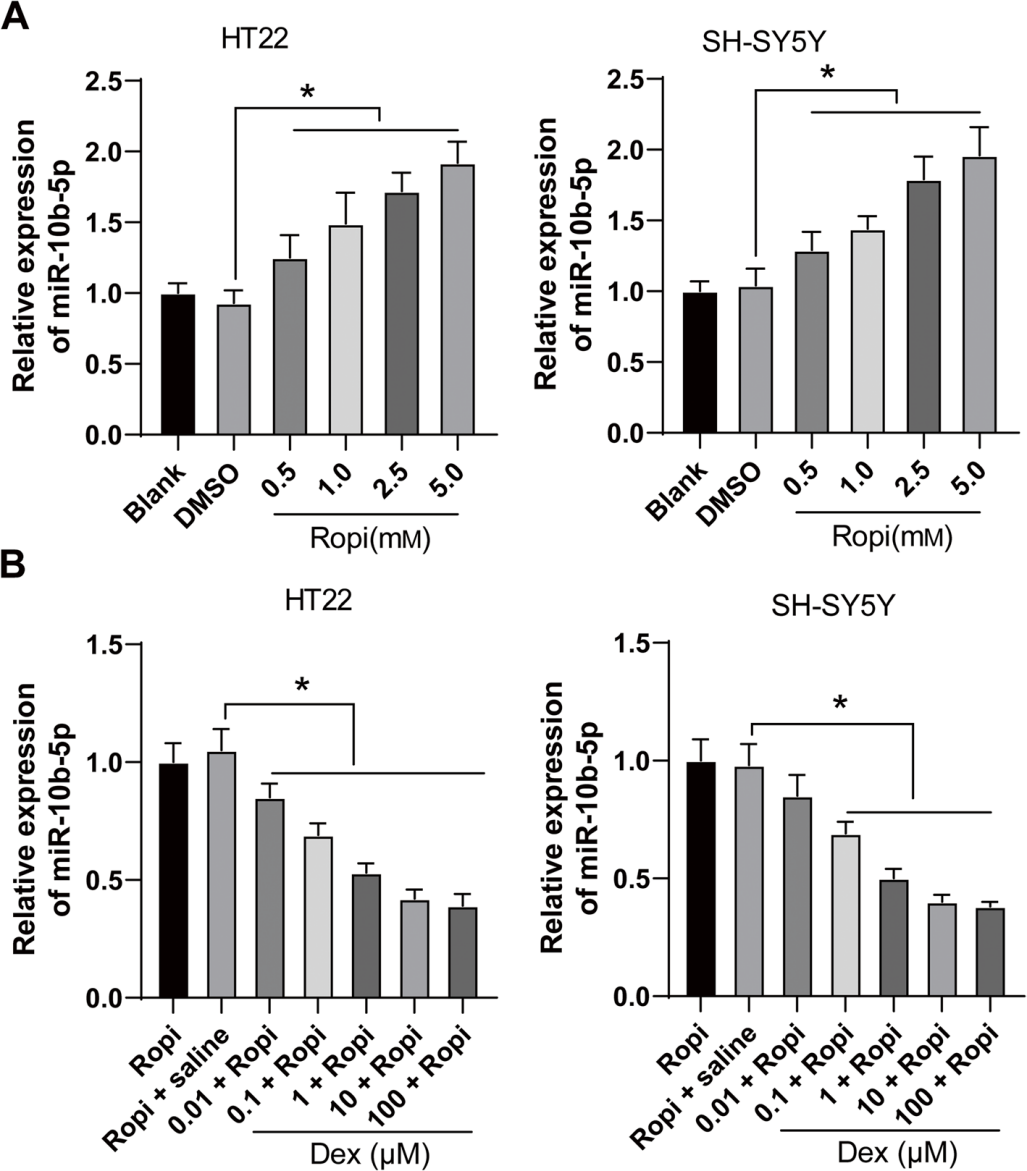
Dexmedetomidine pretreatment suppressed miR-10b-5p expression. **A-B** miR-10b-5p expression in HT22 and SH-SY5Y cells under different treatments was detected using RT-qPCR. The cell experiment was repeated 3 times independently. Data are presented as mean ± standard deviation. Data in panels **A-B** were analyzed using one-way ANOVA, followed by Tukey's multiple comparisons test, ^*^p < 0.05

Then, the mechanism of DEX pretreatment reducing ropivacaine-induced neurotoxicity was explored. miR-10b-5p is highly expressed in hippocampal tissues of rats with stroke and Alzheimer's disease [19, 26]. Hence, we speculated that miR-10b-5p was related to ropivacaine-induced neurotoxicity and DEX protected ropivacaine-induced neurotoxicity by regulating miR-10b-5p expression. RT-qPCR results demonstrated that miR-10b-5p expression in ropivacaine-treated HT22 and SH-SY5Y cells was evidently elevated, and 2.5 mM ropivacaine led to the highest miR-10b-5p expression (*p* < 0.05, Fig. 2A). DEX pretreatment significantly inhibited miR-10b-5p expression, and 10 μM DEX produced the most significant inhibitory effect (*p* < 0.05, Fig. 2B). Briefly, DEX pretreatment suppressed miR-10b-5p expression.

### miR-10b-5p overexpression reversed the protection of DEX pretreatment on ropivacaine-induced neurotoxicity

**Fig. 3:**
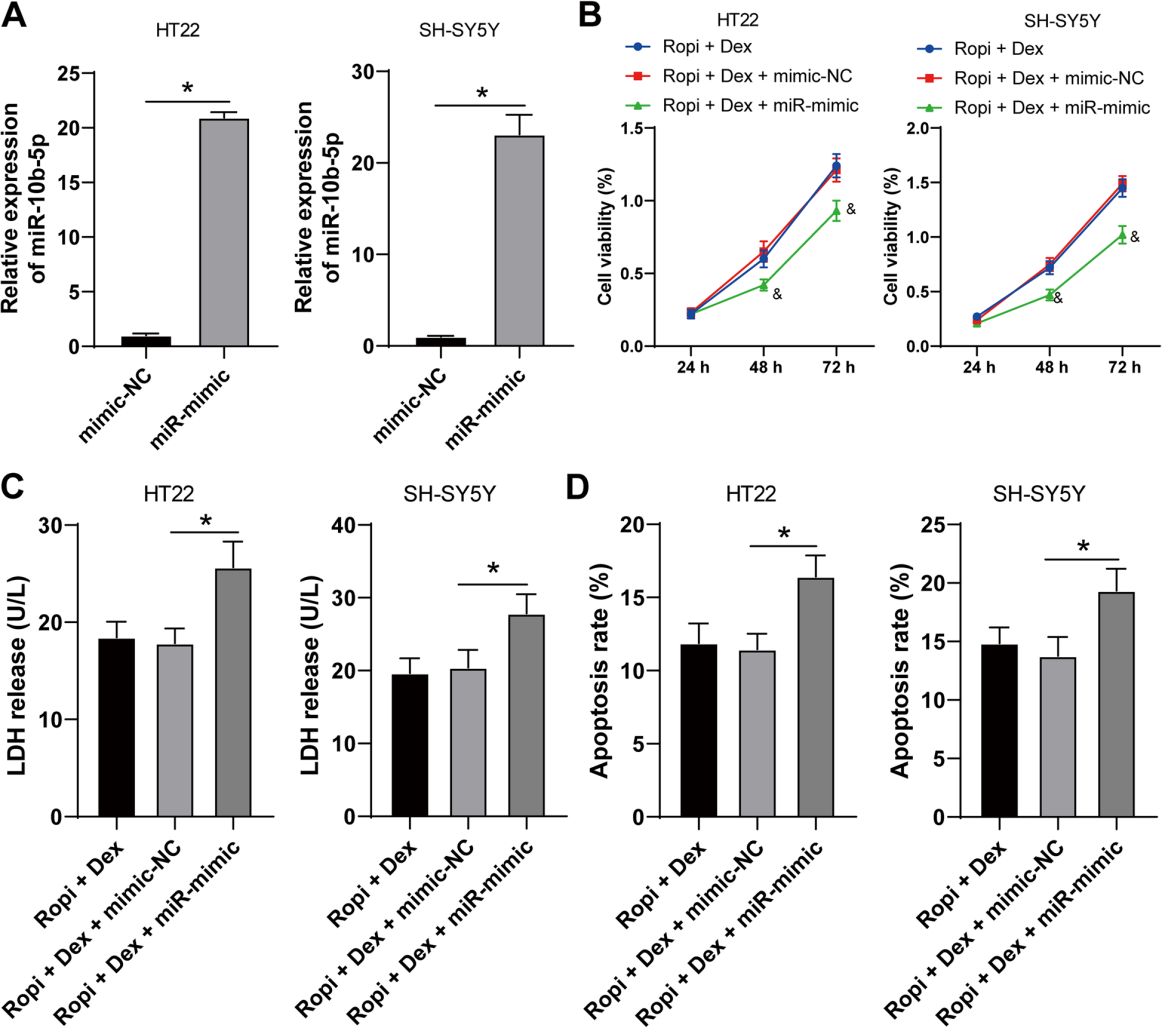
miR-10b-5p overexpression reversed the protection of dexmedetomidine pretreatment on ropivacaine-induced neurotoxicity. miR-10b-5p-mimic (miR-mimic) was transfected into HT22 and SH-SY5Y cells, with mimic-NC as negative control. **A** Transfection efficiency was measured using RT-qPCR, followed by a combined experiment with Dex-pretreated cells. **B** Cell viability was measured using CCK-8 assay. **C** LDH release was measured using LDH detection kit. **D** Apoptosis was measured using flow cytometry. The cell experiment was repeated 3 times independently. Data are presented as mean ± standard deviation. Data in panel **A** were analyzed using t test; data in panel **B** were analyzed using two-way ANOVA, and data in panels **C-D** were analyzed using one-way ANOVA, followed by Tukey's multiple comparisons test, ^&^*p* < 0.05, compared with the Ropi + DEX group, ^*^p < 0.05

Thereafter, we verified whether DEX pretreatment protected ropivacaine-induced neurotoxicity by regulating miR-10b-5p. HT22 and SH-SY5Y cells were transfected with miR-10b-5p-mimic (*p* < 0.05, Fig. 3A), followed by combined treatment with DEX pretreatment. miR-10b-5p overexpression notably reduced the viability of HT22 and SH-SY5Y cells (*p* < 0.05, Fig. 3B), increased LDH release (*p* < 0.05, Fig. 3C), and elevated apoptosis rate (*p* < 0.05, Fig. 3D, Supplementary Fig. 2). These results demonstrated that miR-10b-5p overexpression reversed the protection of DEX pretreatment on ropivacaine-induced neurotoxicity.

### miR-10b-5p targeted BDNF

**Fig. 4:**
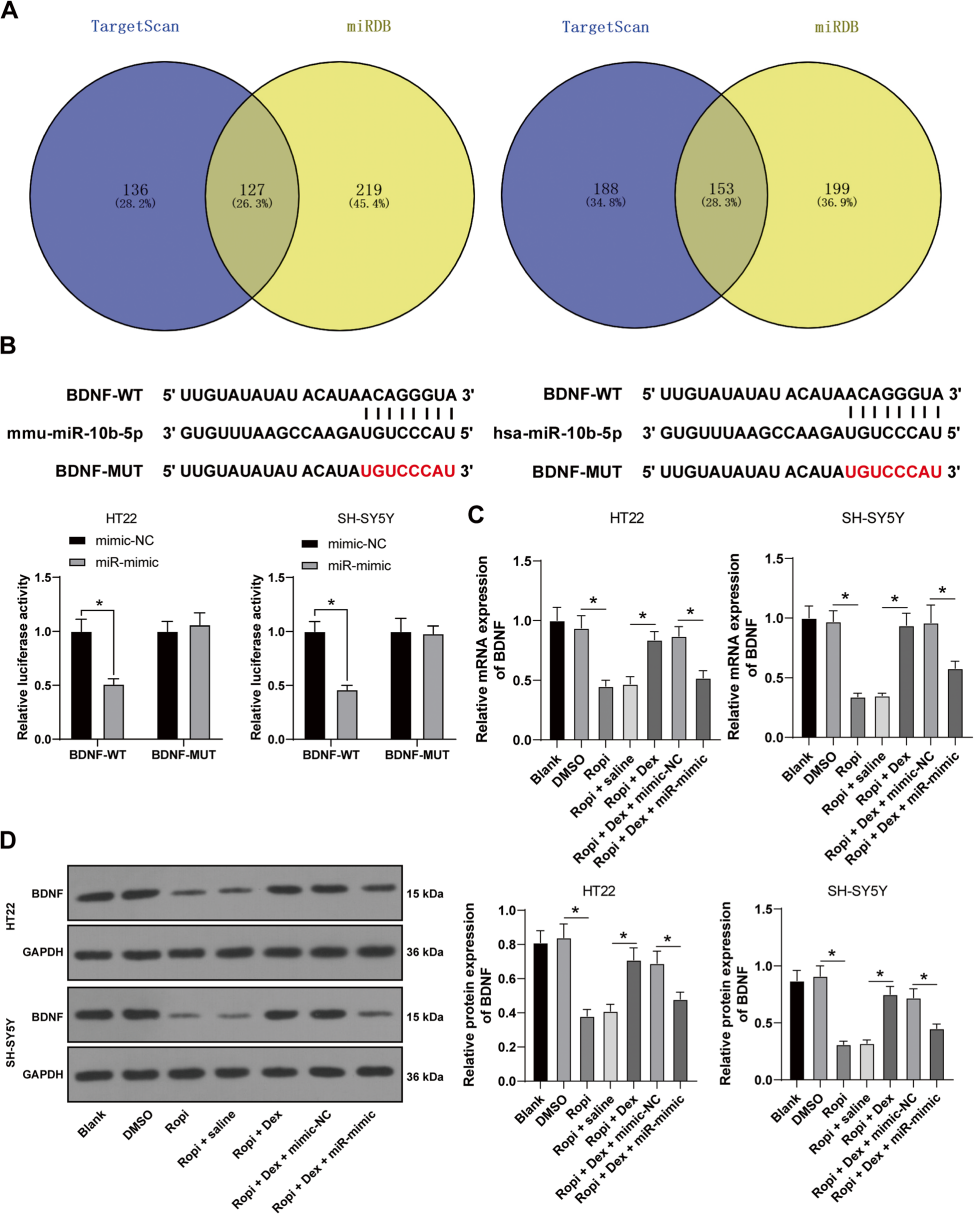
BDNF was the downstream target of miR-10b-5p. **A** The downstream targets of miR-10b-5p were predicted and screened through the TargetScan and miRDB database. **B** The binding relationship between miR-10b-5p and BDNF was verified using dual-luciferase assay. **C-D** BDNF expression in HT22 and SH-SY5Y cells was detected using RT-qPCR and Western blot. The cell experiment was repeated 3 times independently. Data are presented as mean ± standard deviation. Data in panel **B** were analyzed using two-way ANOVA, and data in panels **C-D** were analyzed using one-way ANOVA, followed by Tukey's multiple comparisons test, ^*^p < 0.05

Next, the downstream mechanism of miR-10b-5p was investigated. The downstream targets of miR-10b-5p were predicted and screened through the TargetScan and miRDB databases (Fig. 4A), among which we focused on BDNF. BDNF is poorly expressed in sevoflurane-induced neurotoxicity [27]. Hence, we speculated that BDNF was the target of miR-10b-5p in ropivacaine-induced neurotoxicity. Dual-luciferase assay verified the binding relationship between miR-10b-5p and BDNF (*p* < 0.05, Fig. 4B). RT-qPCR and Western blot results revealed that BDNF expression was decreased in ropivacaine-induced HT22 and SH-SY5Y cells but increased after DEX pretreatment, while miR-10b-5p overexpression notably reduced BDNF expression (*p* < 0.05, Fig. 4C-D). Briefly, miR-10b-5p targeted BDNF expression.

### BDNF silencing reversed the protection of DEX pretreatment on ropivacaine-induced neurotoxicity

**Fig. 5:**
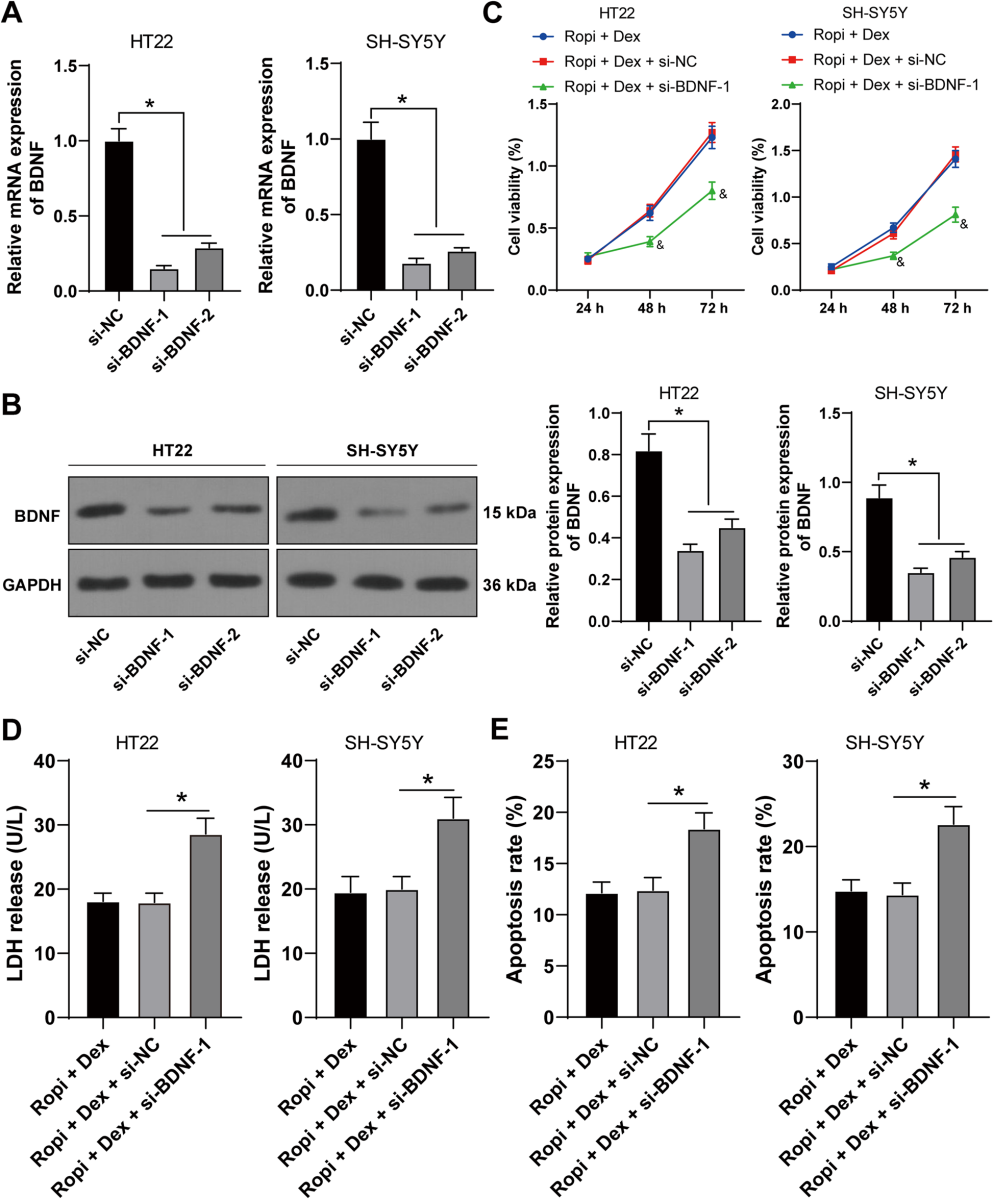
BDNF silencing reversed the protection of dexmedetomidine pretreatment on ropivacaine-induced neurotoxicity. si-BDNF-1 and si-BDNF-2 were transfected into HT22 and SH-SY5Y cells, with si-NC as negative control. **A-B** Transfection efficiency was measured using RT-qPCR and Western blot. si-BDNF-1 with a better silencing effect was selected for combined experiment with Dex-pretreated cells. **C** Cell viability was measured using CCK-8 assay. **D** LDH release was measured using LDH detection kit. **E** Apoptosis was measured using flow cytometry. The cell experiment was repeated 3 times independently. Data are presented as mean ± standard deviation. Data in panel **C** were analyzed using two-way ANOVA, and data in panels **A-B/D-E** were analyzed using one-way ANOVA, followed by Tukey's multiple comparisons test, ^&^*p* < 0.05, compared with the Ropi + DEX group, ^*^p < 0.05

Finally, we further investigated whether BDNF was involved in the protection of DEX pretreatment on ropivacaine-induced neurotoxicity. si-BDNF-1 and si-BDNF-2 were transfected into HT22 and SH-SY5Y cells (*p* < 0.05, Fig. 5A-B). si-BDNF-1 with a better inhibitory effect was used for a combined experiment with DEX pretreatment. BDNF silencing notably reduced the viability of HT22 and SH-SY5Y cells (*p* < 0.05, Fig. 5C), increased LDH release (*p* < 0.05, Fig. 5D), and elevated apoptosis rate (*p* < 0.05, Fig. 5E, Supplementary Fig. 3). Briefly, BDNF silencing reversed the protection of DEX pretreatment on ropivacaine-induced neurotoxicity.

## Discussion

It has been recognized that ropivacaine can cause neurotoxicity during the perioperative period, especially at a high concentration and/or for prolonged use [6]. DEX, a kind of α2 adrenergic receptor agonist, has been commonly used as an adjuvant of ropivacaine and has shown neuroprotective properties in multiple experimental models [28–30]. To the best of our knowledge, our study is the first-of-its-kind to demonstrate that DEX pretreatment alleviated ropivacaine-induced neurotoxicity via the miR-10b-5p/BDNF axis.

Excessive use of ropivacaine impairs the central nervous system and causes neurotoxicity in the perioperative period of local anesthesia effects [31]. Seizures induced by ropivacaine are related to hippocampal neurons [32]. Therefore, we deemed that excessive use of ropivacaine affected the central nervous system and was related to the hippocampus. In the present study, we selected the hippocampal neurons HT22 and SH-SY5Y cell lines to test our hypothesis. HT22 cell is a kind of mouse hippocampal neuron, which is widely used for an in vitro neuron model related to neurotoxicity [33]. SH-SY5Y is a neural cell line widely used in neurotoxicity research, and its toxicity sensitivity is similar to that of primary neuronal cells. SH-SY5Y cells have been commonly used for the establishment of a cell model of ropivacaine-induced neurotoxicity [34]. The concentrations of ropivacaine and DEX used in this study were determined according to the previous literature reports [35–37]. Specifically, HT22 and SH-SY5Y cells were subjected to ropivacaine insult (0.5 mM, 1 mM, 2.5 mM, and 5 mM) for reproducing its damage characteristics. Then, the cells were pretreated with different concentrations of DEX (0.01 μM, 0.1 μM, 1 μM, 10 μM, and 100 μM). The clinical doses of ropivacaine are 0.5% and 1% [38], about 1.8 mM and 3.6 mM. The clinical doses of DEX are 2.7 μM, 11.7 μM, and 34.1 μM [39]. The concentrations of ropivacaine and DEX used in our in vitro experiments were similar to the clinically relevant doses. The concentrations of ropivacaine (2.5 mM) and DEX (10 μM) with optimal effects were screened by CCK-8 assay. The results showed that the viability of HT22 cells and SH-SY5Y cells was decreased with the increase of ropivacaine concentration. The cell viability reached the lowest when the concentration of ropivacaine was 2.5 mM, but no longer decreased with the increase of ropivacaine concentration. Hence, we used 2.5 mM ropivacaine for subsequent experimentation. As a key enzyme of anaerobic metabolism, LDH released from degenerated neurons can reflect the degree of nerve injury [40]. Accumulating studies have confirmed that neurotoxicity induced by local anesthetics is related to apoptosis [36, 41, 42]. Local anesthetics induce the activation of apoptotic neuronal cell death and finally determine the degree of nerve injury [43]. Our results showed that ropivacaine treatment elevated LDH release in HT22 and SH-SY5Y cells and enhanced the cell apoptosis, suggesting that ropivacaine treatment induced neurotoxicity, which is consistent with the relevant results of anesthesia-induced neurotoxicity reported in the previous literature [44]. DEX has wide application in the clinic as an adjuvant to local anesthetics, which can ameliorate neuronal injury and improve functional outcomes in some preclinical models of anesthetic-induced neurotoxicity [11]. Also, DEX pretreatment has been demonstrated to attenuate propofol-induced neurotoxicity in neurons from the rat hippocampus [37]. Consistently, our results revealed that DEX pretreatment notably reduced LDH release and repressed cell apoptosis. DEX combined with ropivacaine potently alleviates postoperative pain and improves cognitive function in patients receiving craniocerebral surgery [30]. DEX combined with ropivacaine not only prolongs the sensory and motor block duration of sciatic nerve in rats but also mitigates ropivacaine-induced neurotoxicity by suppressing caspase-3-dependent apoptosis of sciatic nerve cells [12]. Accordingly, we found that DEX pretreatment relieved ropivacaine-induced neurotoxicity in HT22 and SH-SY5Y cells, as evidenced by reduced LDH release and suppressed apoptosis rate.

Thereafter, we determined the protective mechanism of DEX in ropivacaine-induced neurotoxicity. miR-10b-5p is highly expressed in hippocampal tissues of AD rats, and miR-10b-5p knockdown abates neuronal injury in AD rats [19]. Importantly, DEX has been demonstrated to suppress miR-10b-5p expression, thereby reducing neuronal apoptosis and enhancing neuronal viability in ischemia-anoxia-mediated neurological injury [20]. Hence, we speculated that DEX pretreatment protected HT22 and SH-SY5Y cells from ropivacaine-induced neurotoxicity by regulating miR-10b-5p. Our results demonstrated that miR-10b-5p expression in ropivacaine-treated HT22 and SH-SY5Y cells was dramatically elevated, while DEX pretreatment reversed the aberrant elevation of miR-10b-5p. In functional rescue experiments, miR-10b-5p overexpression led to significantly reduced viability of HT22 and SH-SY5Y cells, elevated LDH release, and enhanced apoptosis, suggesting that miR-10b-5p overexpression reversed the protective effect of DEX on ropivacaine-induced neurotoxicity.

Subsequently, we sought to explore the downstream mechanism of miR-10b-5p in ropivacaine-induced HT22 and SH-SY5Y cells. After transcription, miRNAs interact with the complementary sequences of their target mRNAs at the posttranscriptional level to regulate their expression [14]. The downstream targets of miR-10b-5p were predicted through the databases, in which we focused on BDNF. BDNF, one of the most widely distributed neurotrophins in the mammalian brain, is a critical regulator of neurite growth, synaptic plasticity, and functional neuronal connection selection in the central nervous system [45]. BDNF can stimulate and promote the growth and differentiation of nerve cells and prevent neuronal injury and death [46]. BDNF reduces the apoptosis of neurons submitted to oxygen–glucose deprivation/reoxygenation [47] and promotes neuronal survival after neonatal hypoxic-ischemic encephalopathy [48]. Moreover, the manipulation of the BDNF/TrkB pathway contributes to reversing neuronal apoptosis and alleviating neurotoxicity induced by methamphetamine [23] and propofol [49]. The upregulation of miR-10b-5p results in the decrease of BDNF levels in mouse hippocampal neurogenesis and cognitive impairment model [18]. Based on the above findings, we speculated that DEX pretreatment regulated neuronal viability by upregulating BDNF through miR-10b-5p, thereby preventing neuronal apoptosis and protecting against ropivacaine-induced neurotoxicity. Our results verified that BDNF was the downstream target gene of miR-10b-5p in ropivacaine-induced neurotoxicity. DEX can attenuate sevoflurane-induced neurotoxicity in developing rats [50] and alleviate propofol-induced hippocampal neuronal apoptosis by upregulating BDNF [51]. We found that BDNF was poorly expressed in ropivacaine-induced HT22 and SH-SY5Y cells, while DEX pretreatment notably increased BDNF expression. BDNF represses apoptosis of neurons and alleviates ropivacaine-induced neuronal injury by activating the Akt signaling pathway [52]. Similarly, our results revealed that BDNF silencing reduced HT22 and SH-SY5Y cell viability, enhanced LDH release, and elevated apoptosis rate, indicating that BDNF silencing reversed the protective effect of DEX on ropivacaine-induced neurotoxicity in HT22 and SH-SY5Y cells. Notably, our study found for the first time that miR-10b-5p protected hippocampal neurons in ropivacaine-induced neurotoxicity, and DEX attenuated ropivacaine-induced neurotoxicity by regulating the miR-10b-5p/BDNF axis.

## Conclusions

To conclude, our results suggested that ropivacaine induced neurotoxicity by reducing cell viability, promoting apoptosis, and increasing LDH release, and DEX pretreatment reversed these effects. We also demonstrated that the protection of DEX pretreatment against ropivacaine-induced neurotoxicity was achieved via the miR-10b-5p/BDNF axis. These results may provide a new theoretical basis for DEX in the treatment of ropivacaine-induced neurotoxicity. However, this study also has some limitations. Firstly, we did not verify the protective mechanism of DEX pretreatment in animal models. Secondly, there are many other downstream targets of miR-10b-5p, and we merely selected BDNF for analysis. Thirdly, whether DEX pretreatment can regulate other miRNAs in ropivacaine-induced neurotoxicity remains unclear. In the future, we will investigate more potential miRNA mechanisms of DEX in ropivacaine-induced neurotoxicity, select other downstream targets of miR-10b-5p for analysis, and verify the mechanism of DEX in animal models.

## Supplementary Information

**Additional file 1.**

**Additional file 2: Supplementary Fig. 1.** 

**Additional file 3: Supplementary Fig. 2.** 

**Additional file 4: Supplementary Fig. 3.** 

## Data Availability

The datasets used and/or analysed during the current study are available from the corresponding author on reasonable request.
