# Supplementary figures and images for: Dexmedetomidine pretreatment alleviates ropivacaine-induced neurotoxicity via the miR-10b-5p/BDNF axis
Source: BMC Anesthesiol. 2022 Sep 26;22:304. doi: 10.1186/s12871-022-01810-6 (PMC9511747; doi:10.1186/s12871-022-01810-6)

Figure 4D

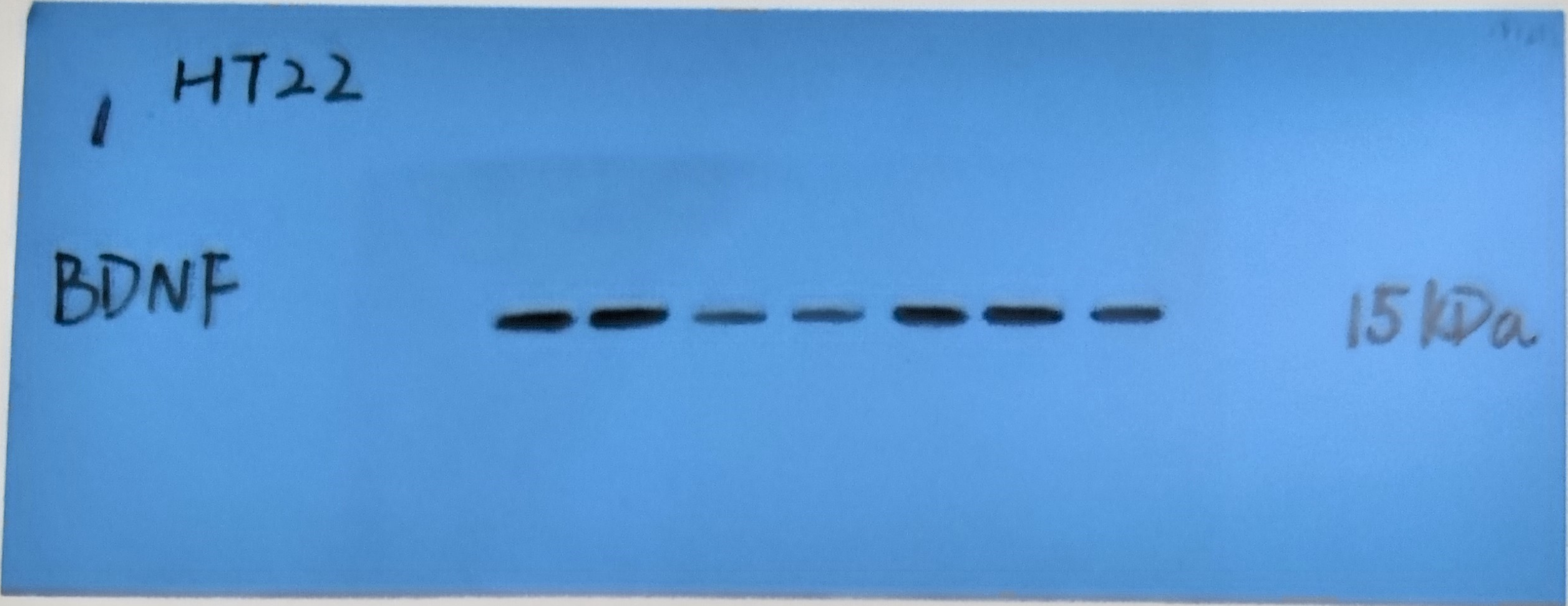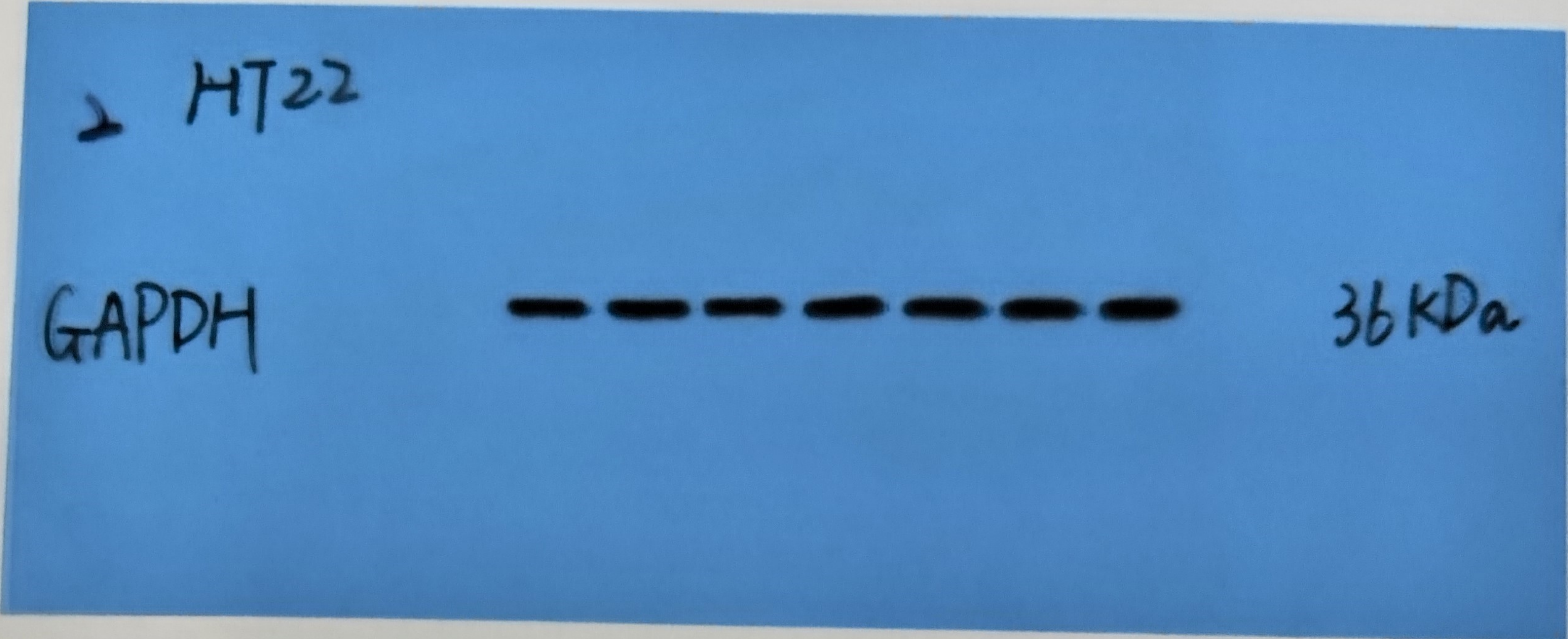

Figure 5B

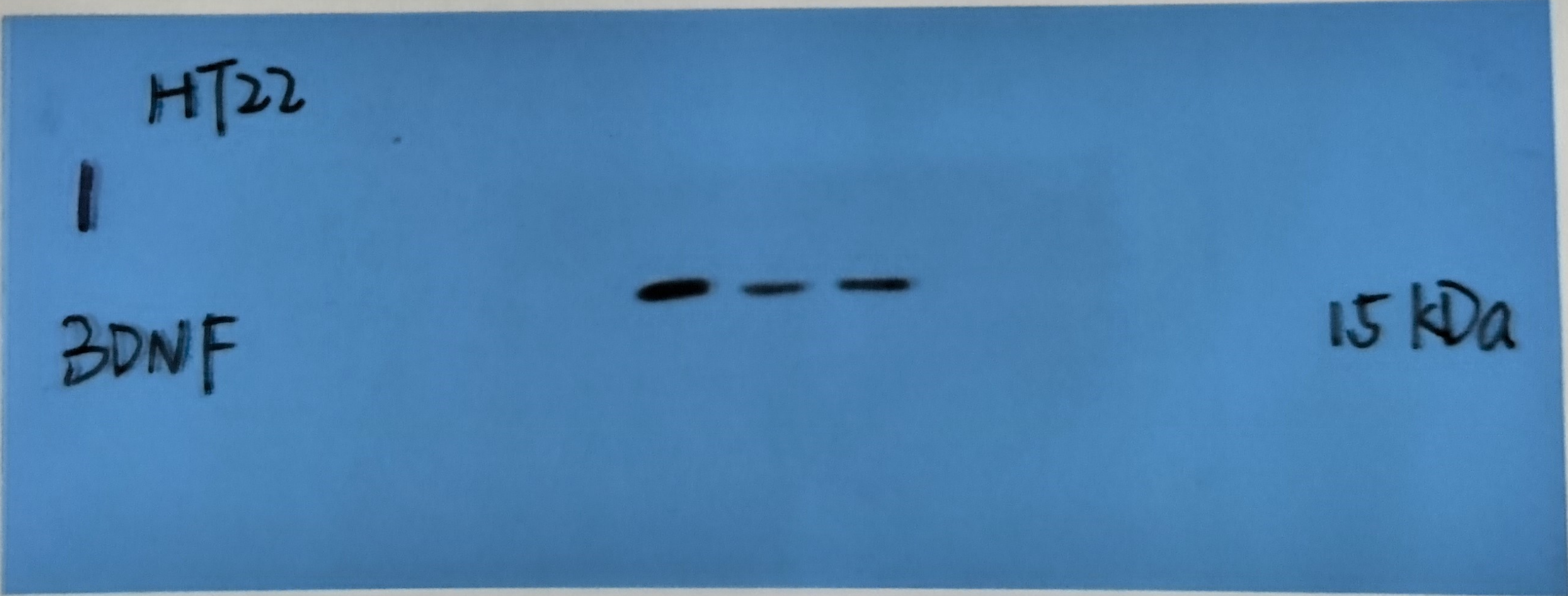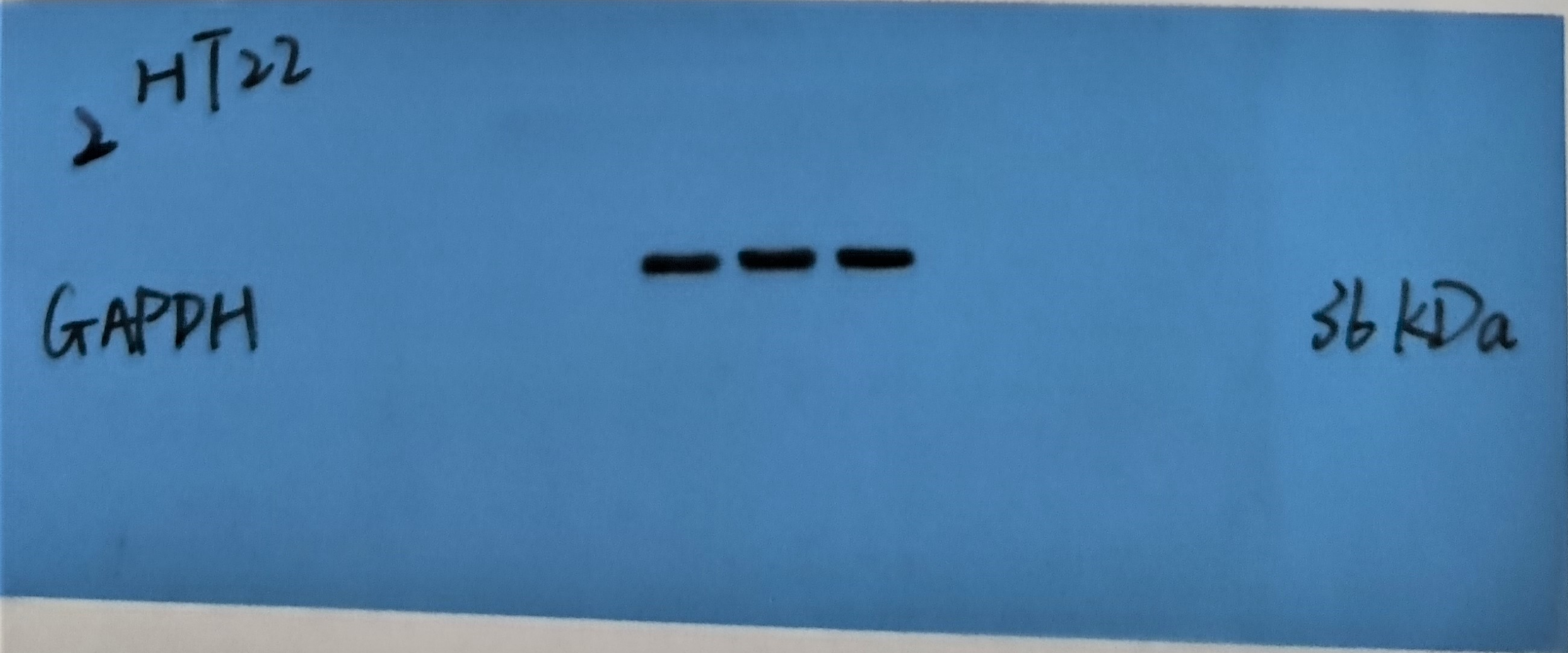

Figure 4D

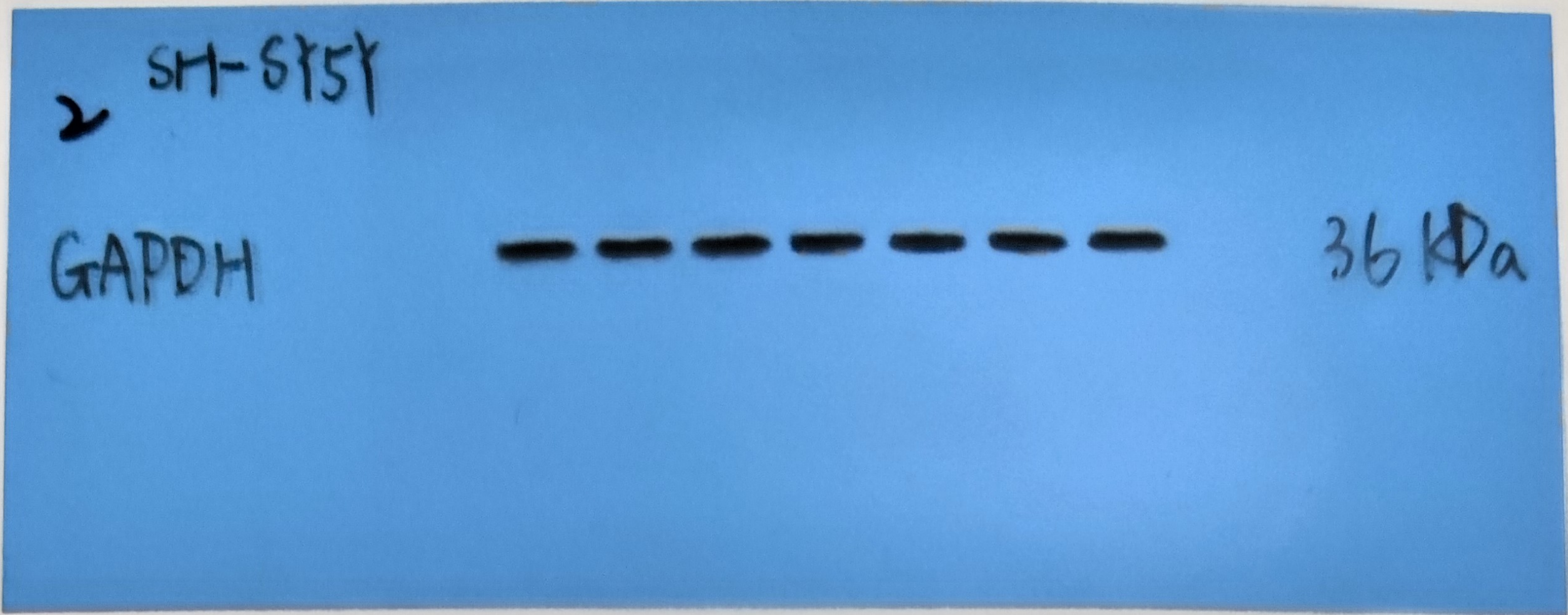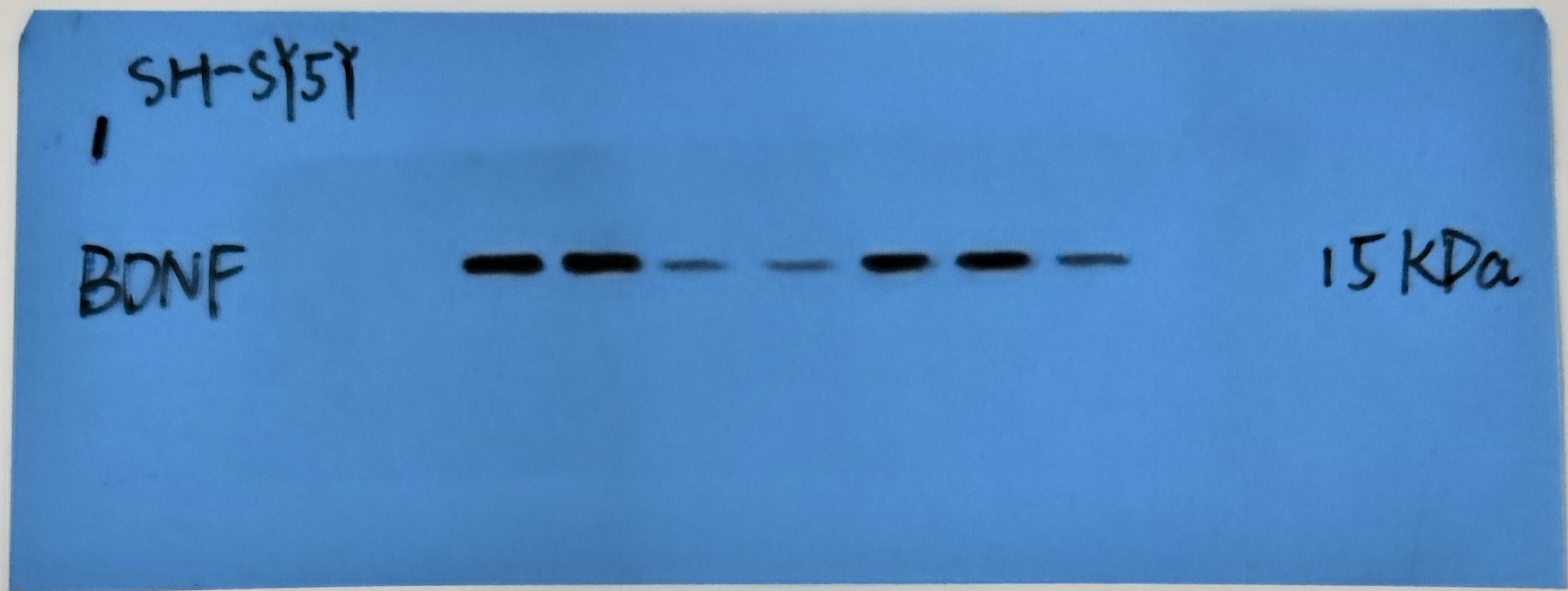

Figure 5B

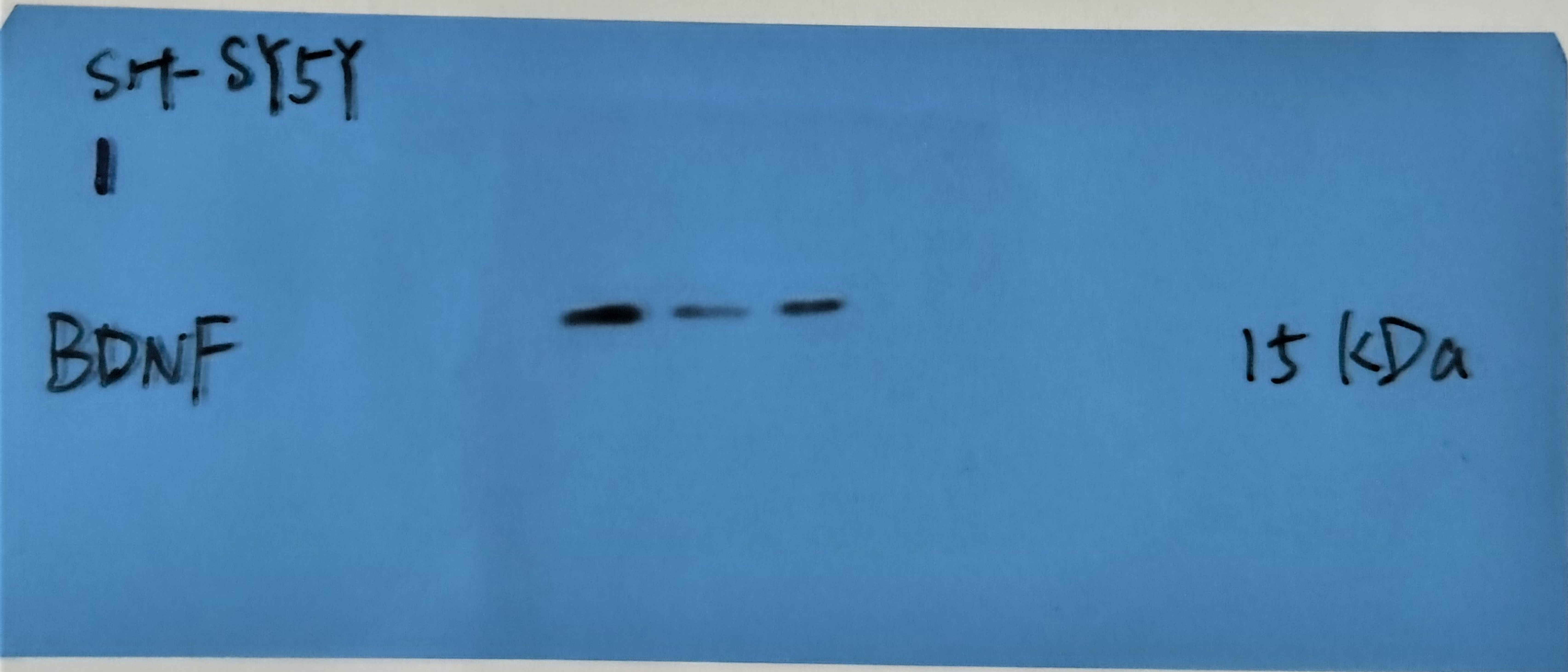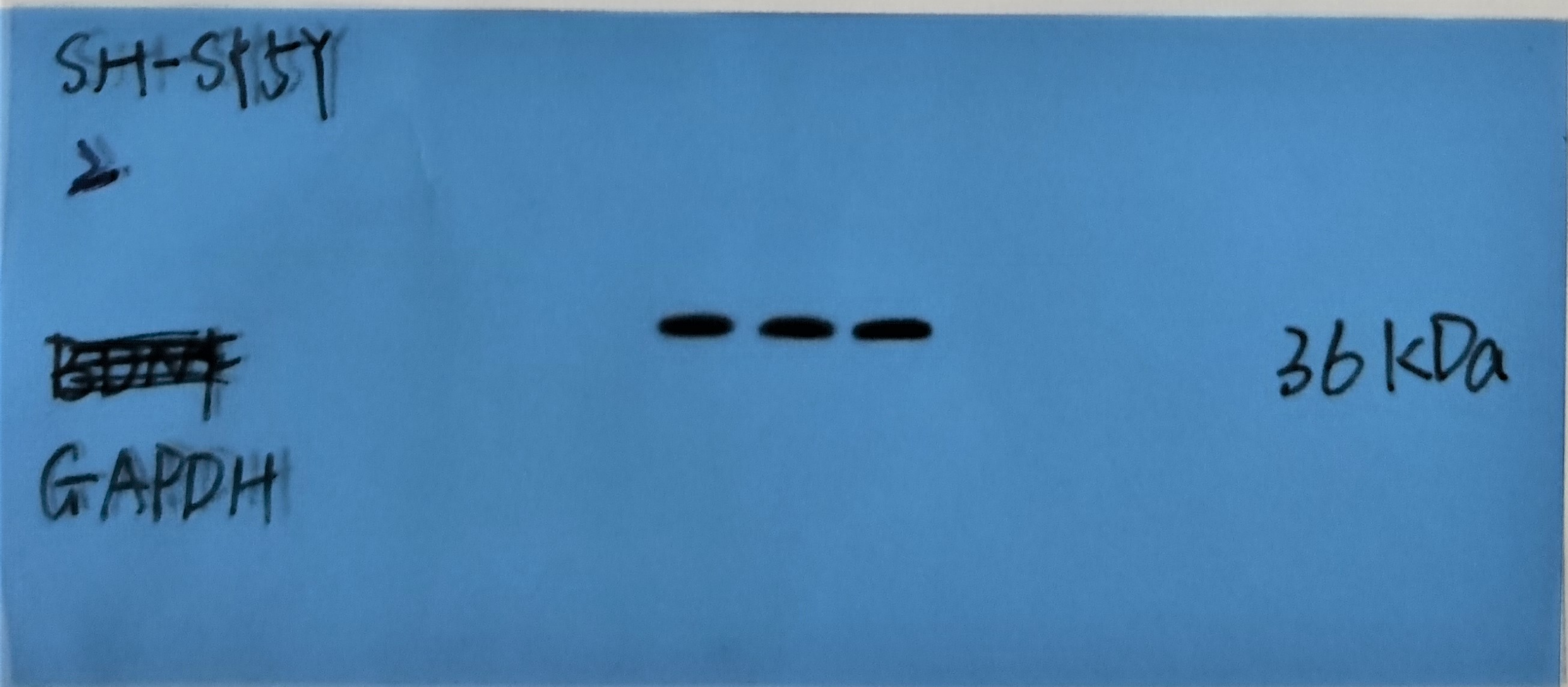

Supplement: Supplementary file 1 — Additional file 1. [file 12871_2022_1810_MOESM1_ESM.zip › WB original data 1.pdf]

Figure 4D

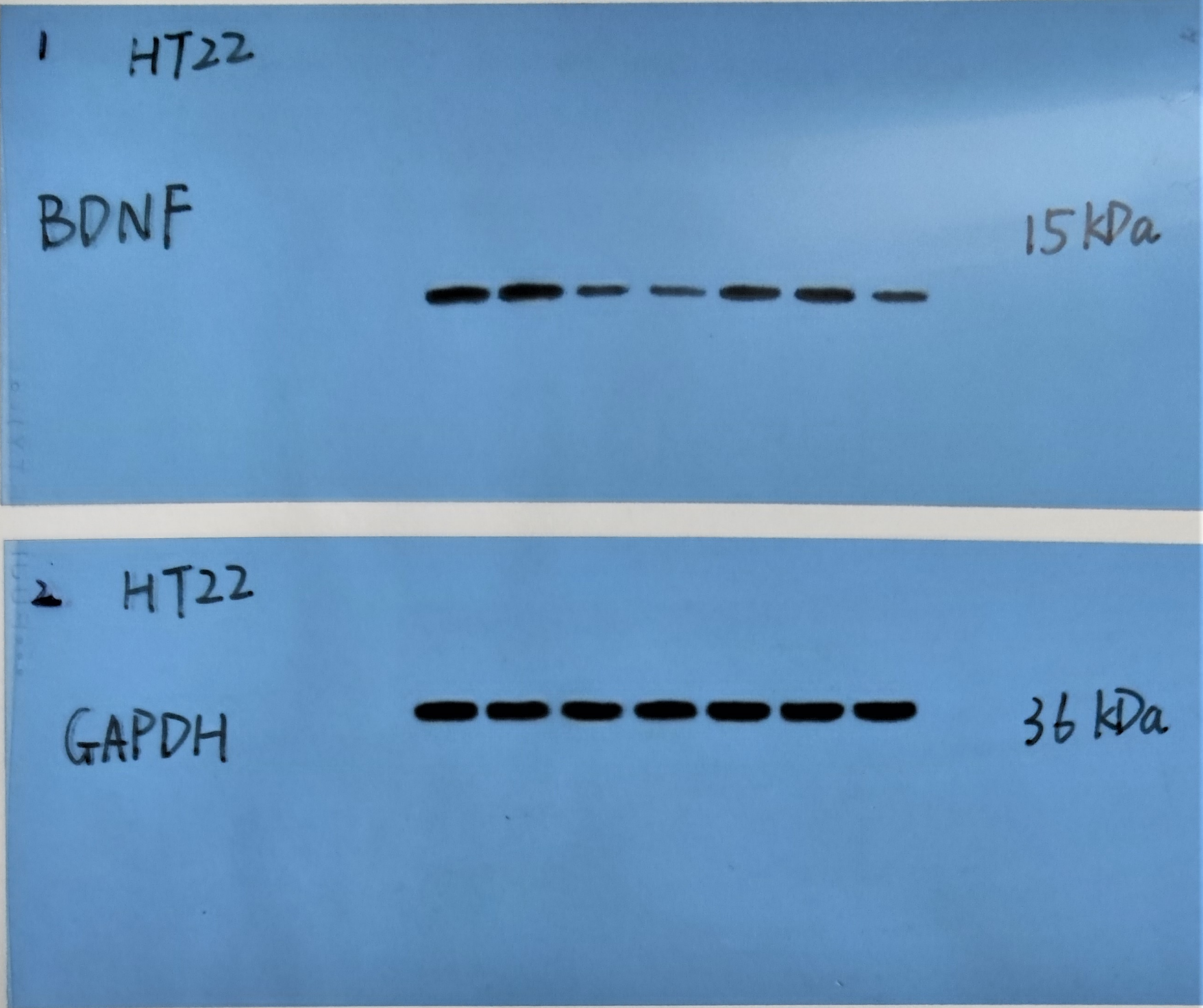

Figure 5B

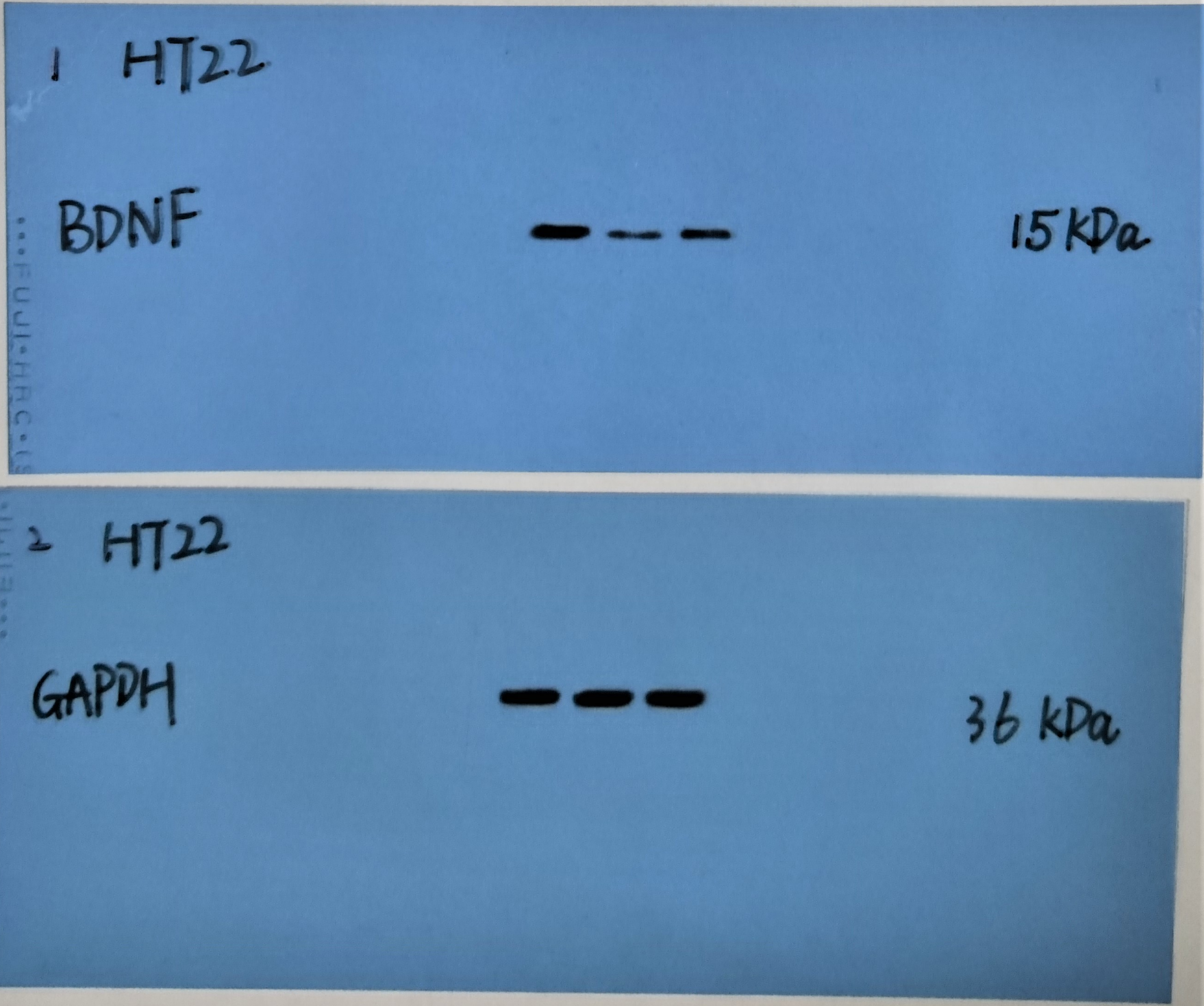

Figure 4D

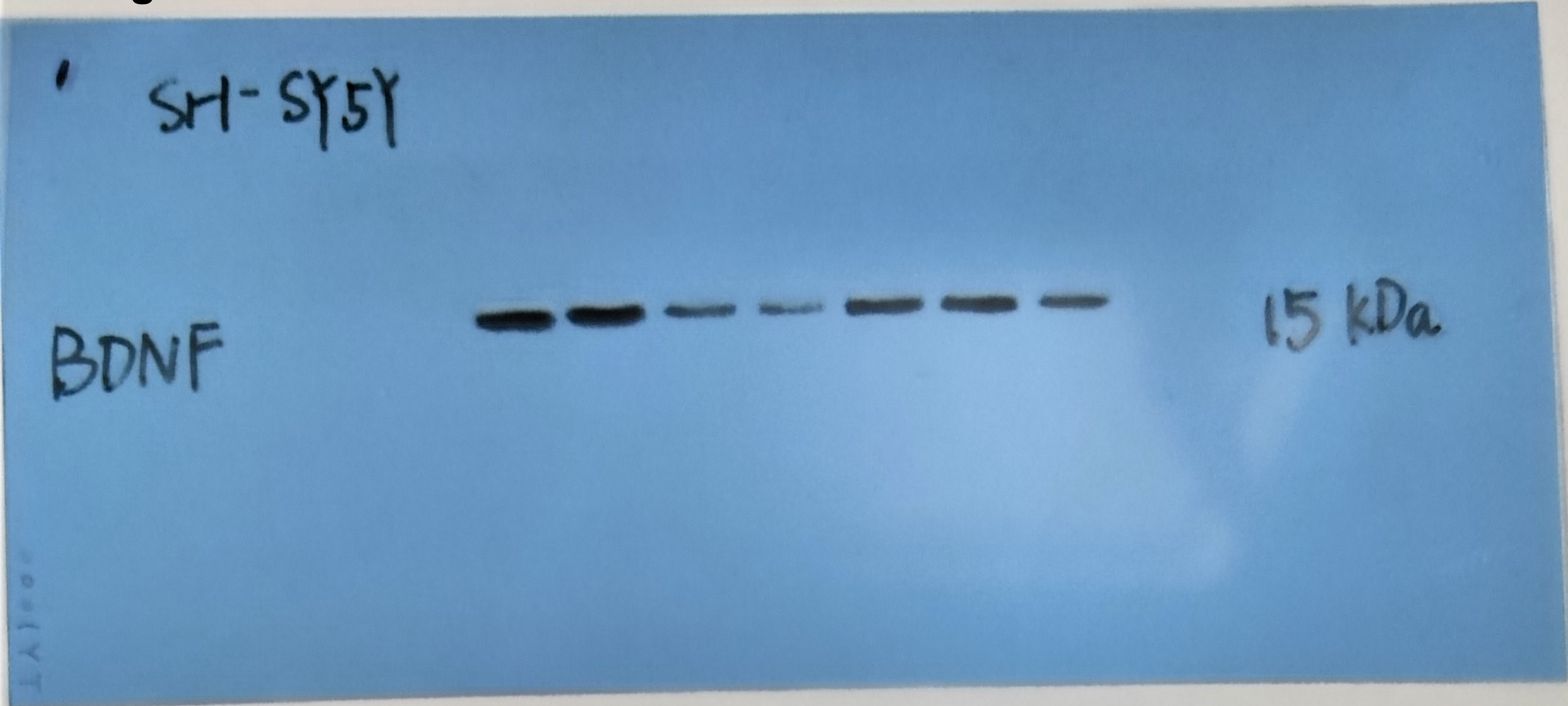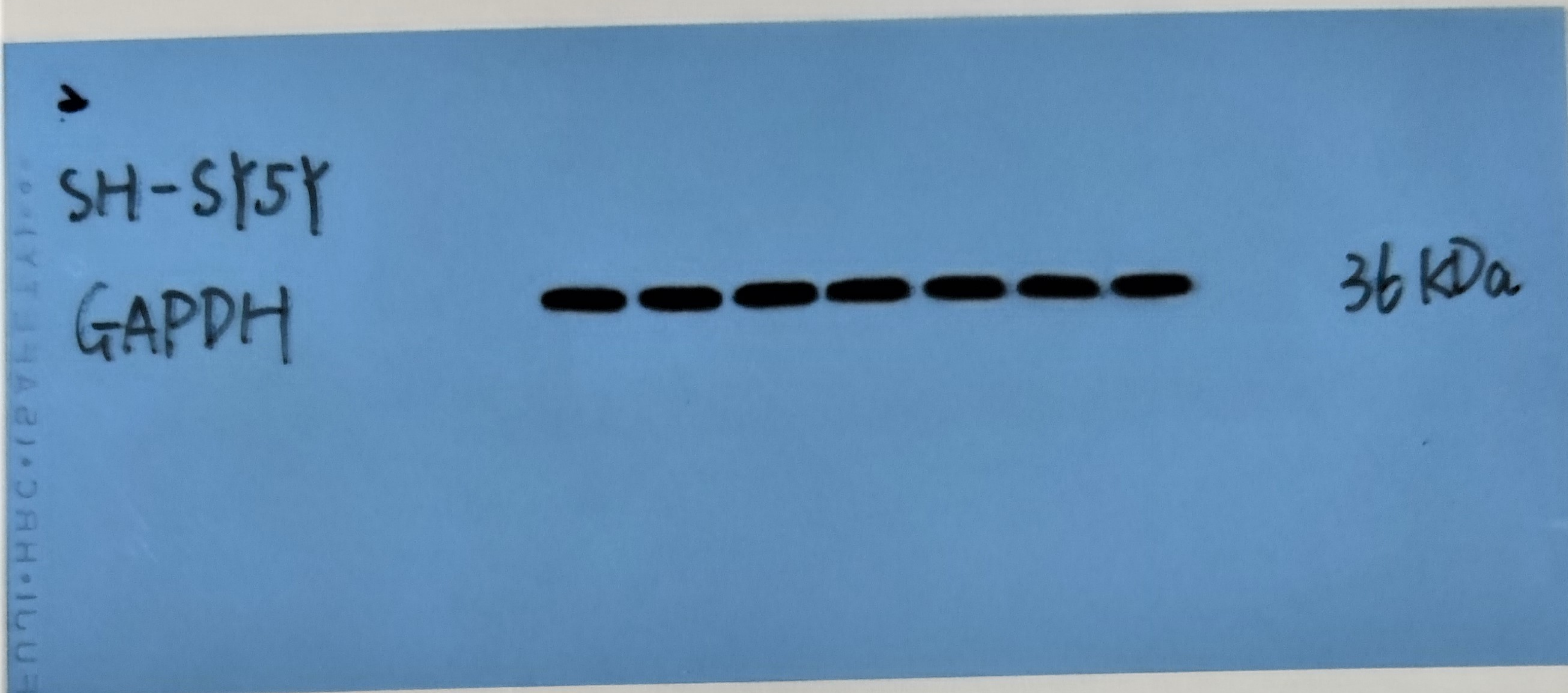

Figure 5B

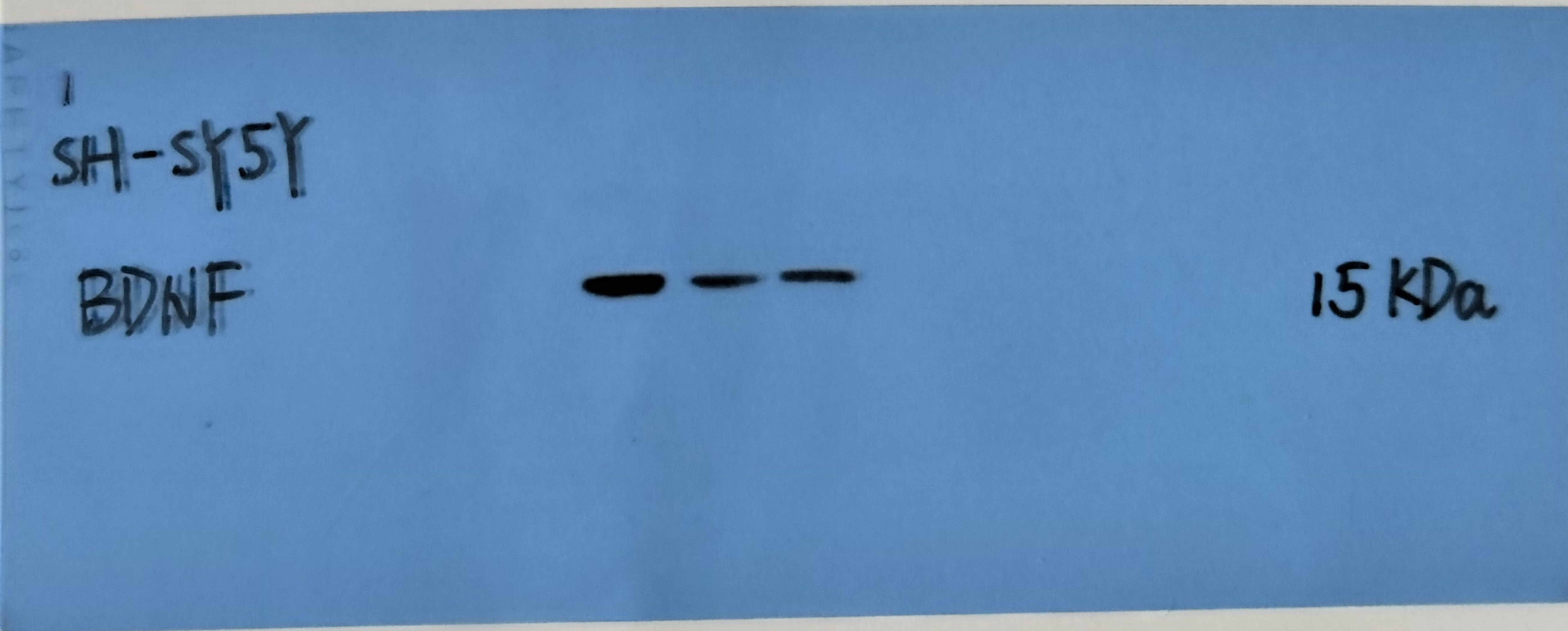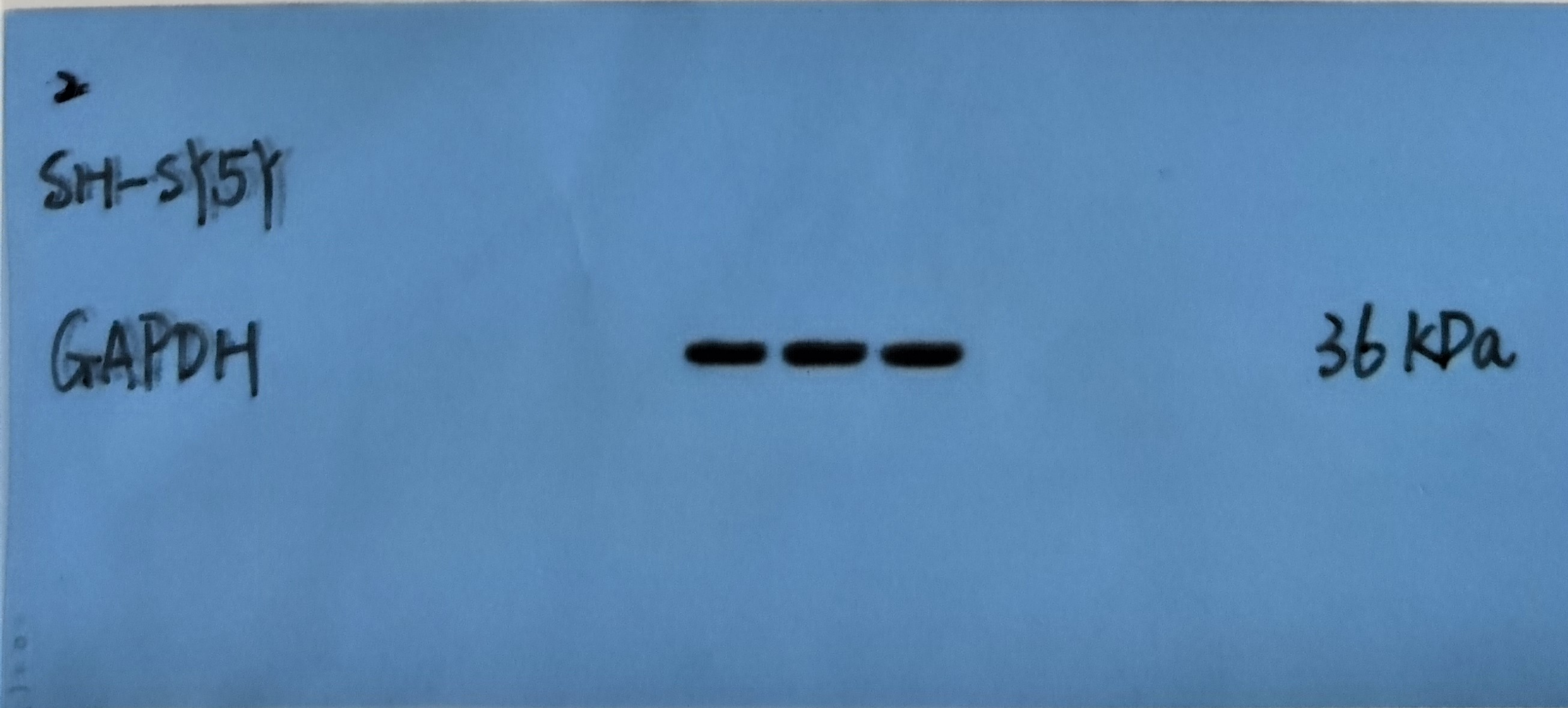

Supplement: Supplementary file 1 — Additional file 1. [file 12871_2022_1810_MOESM1_ESM.zip › WB original data 2.pdf]

Figure 4D

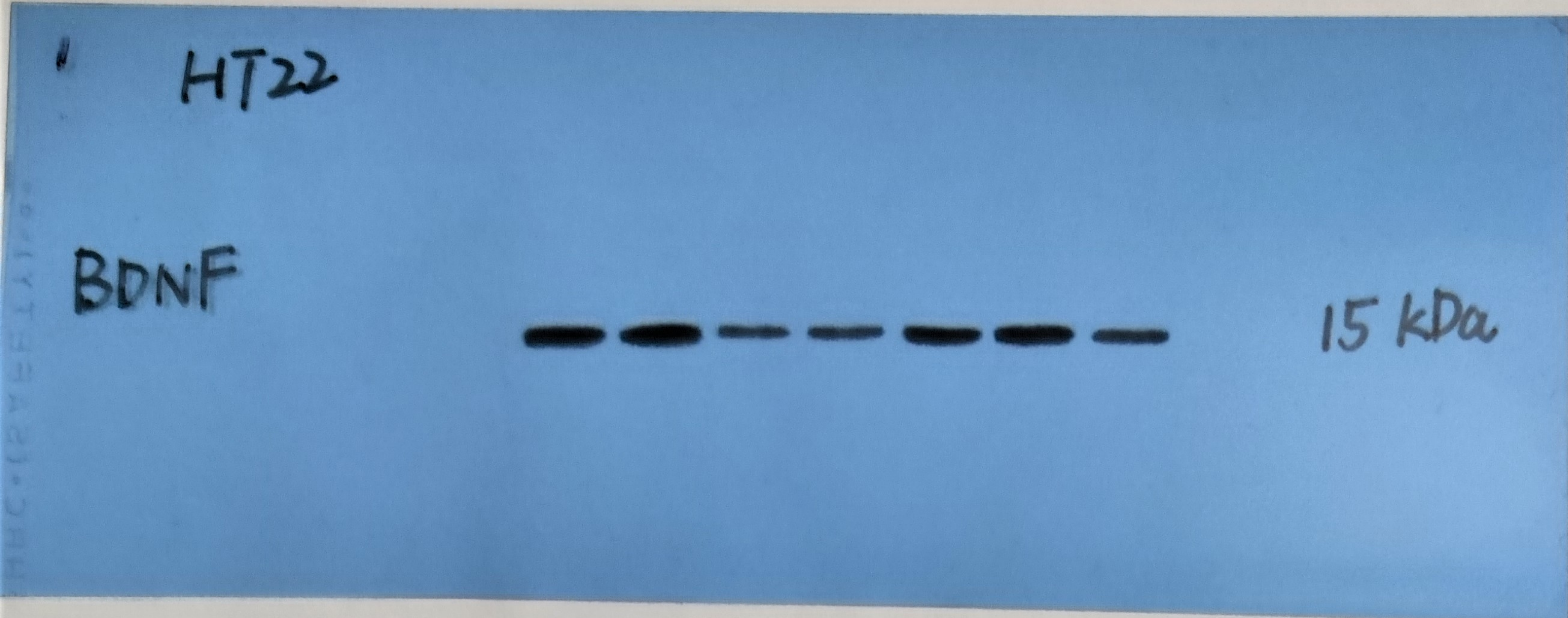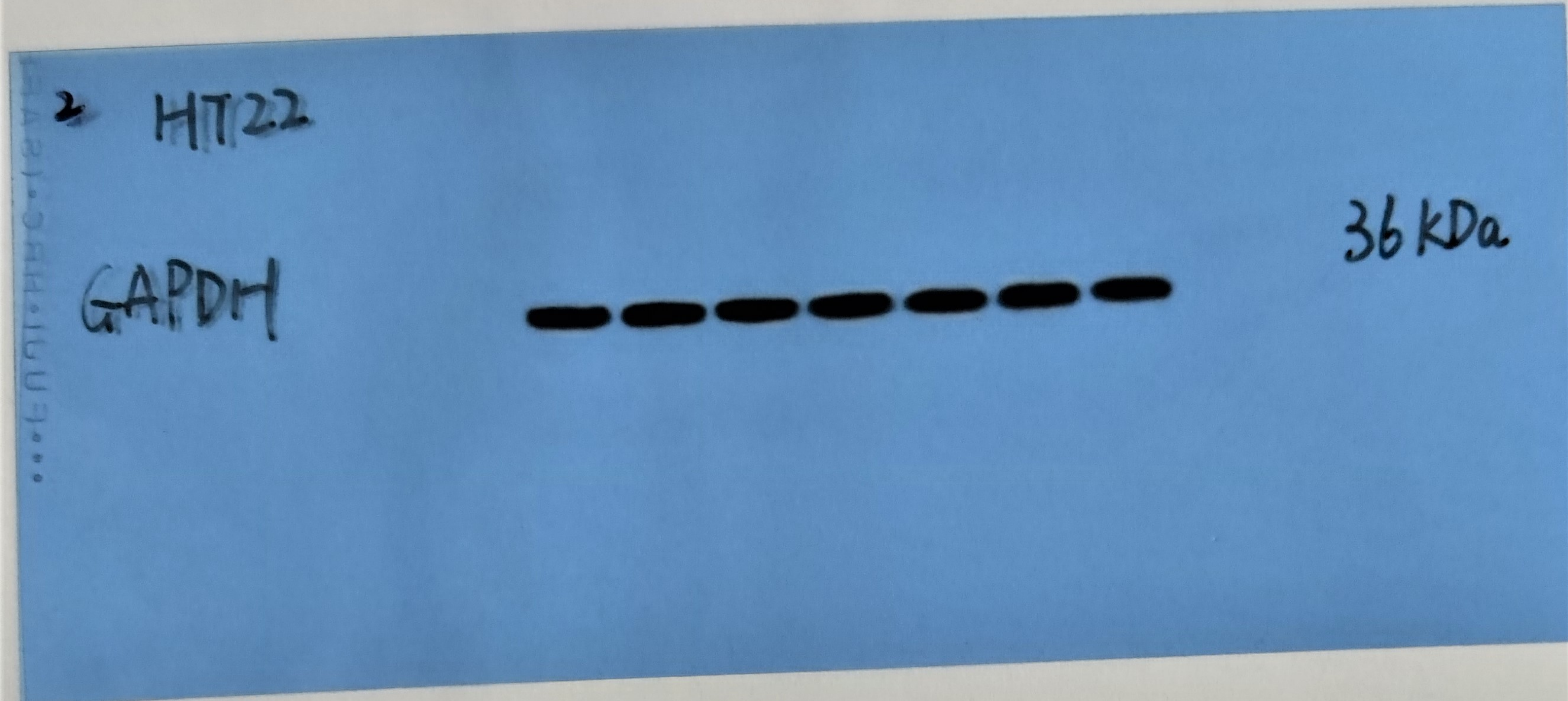

Figure 5B

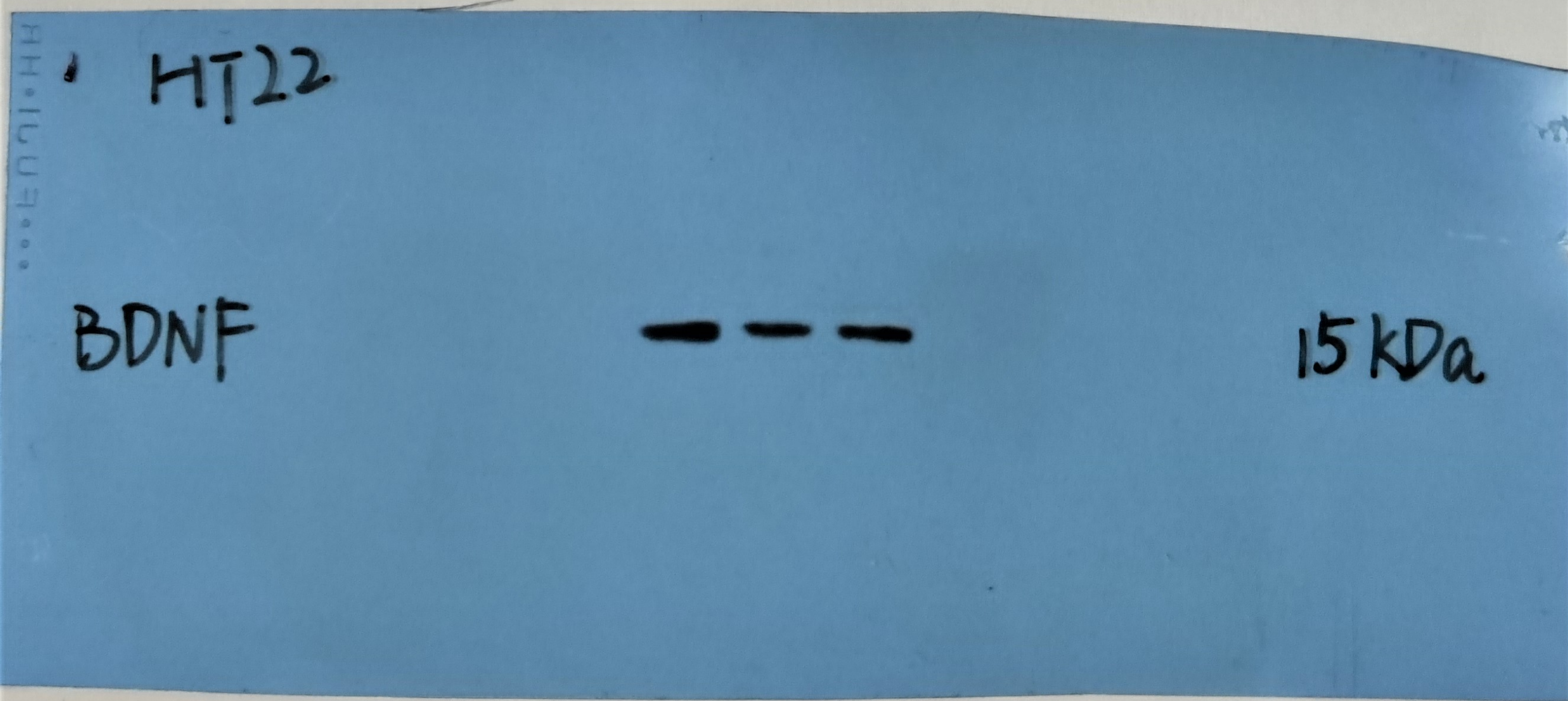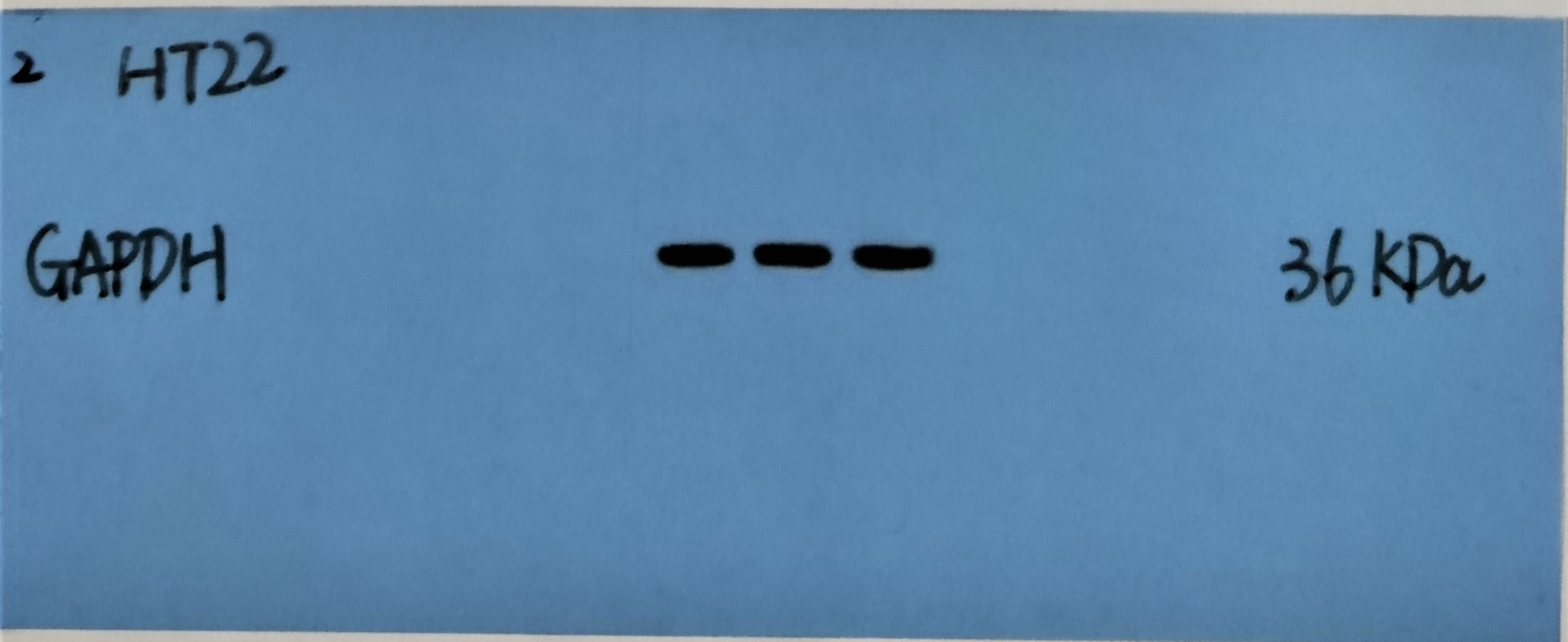

Figure 4D

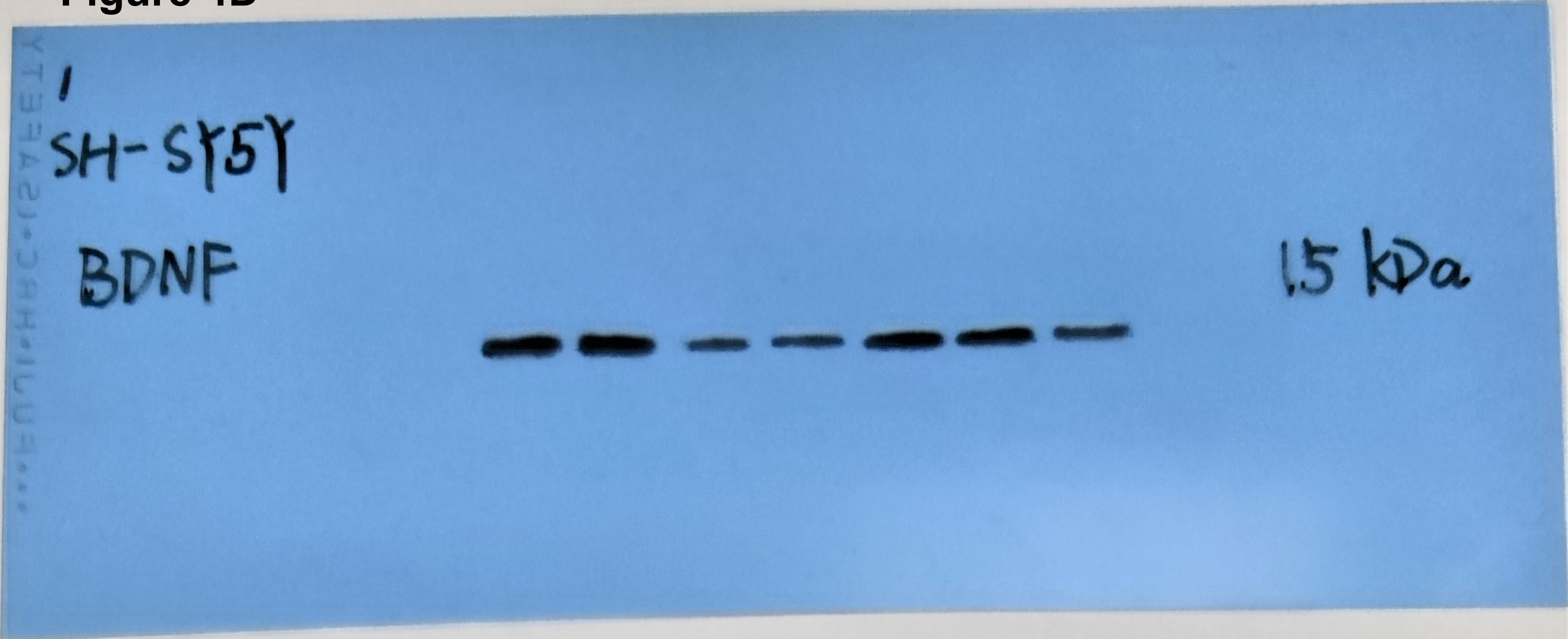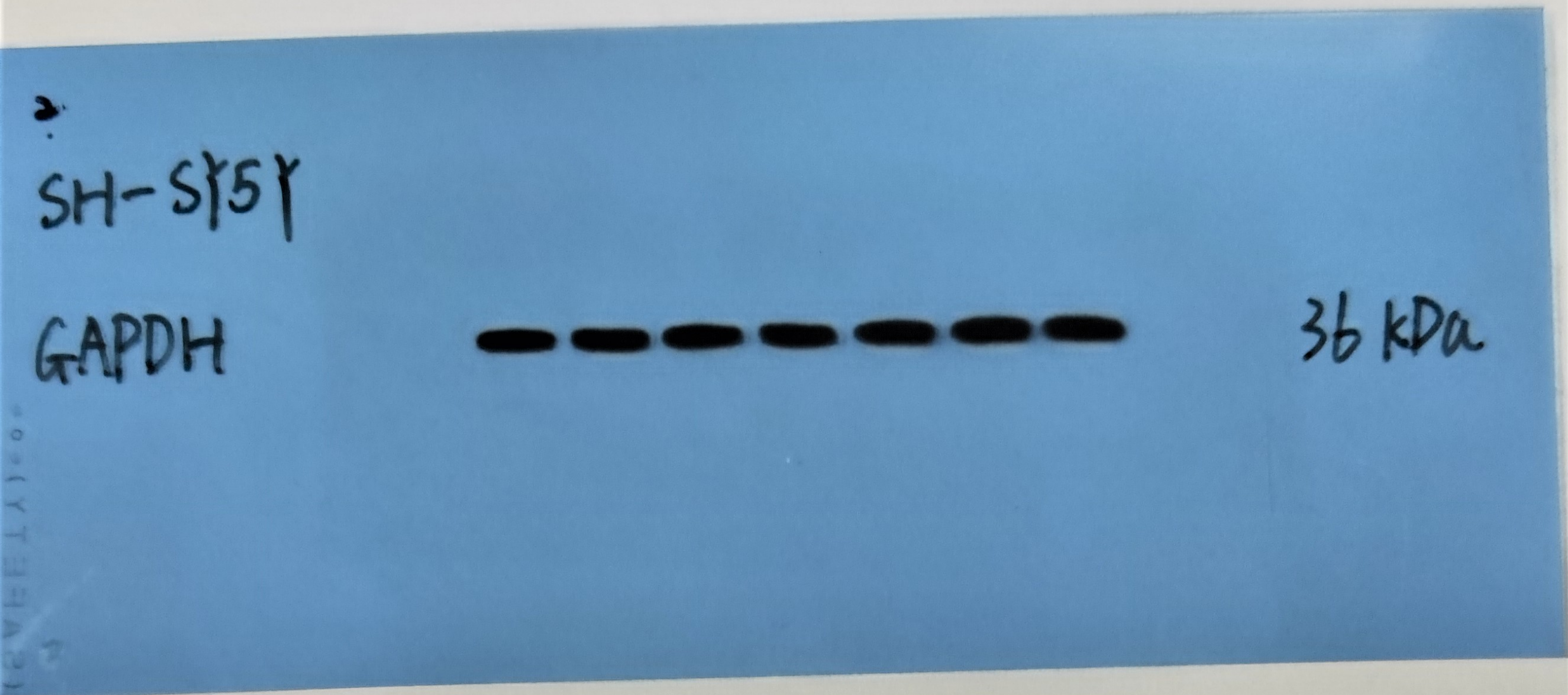

Figure 5B

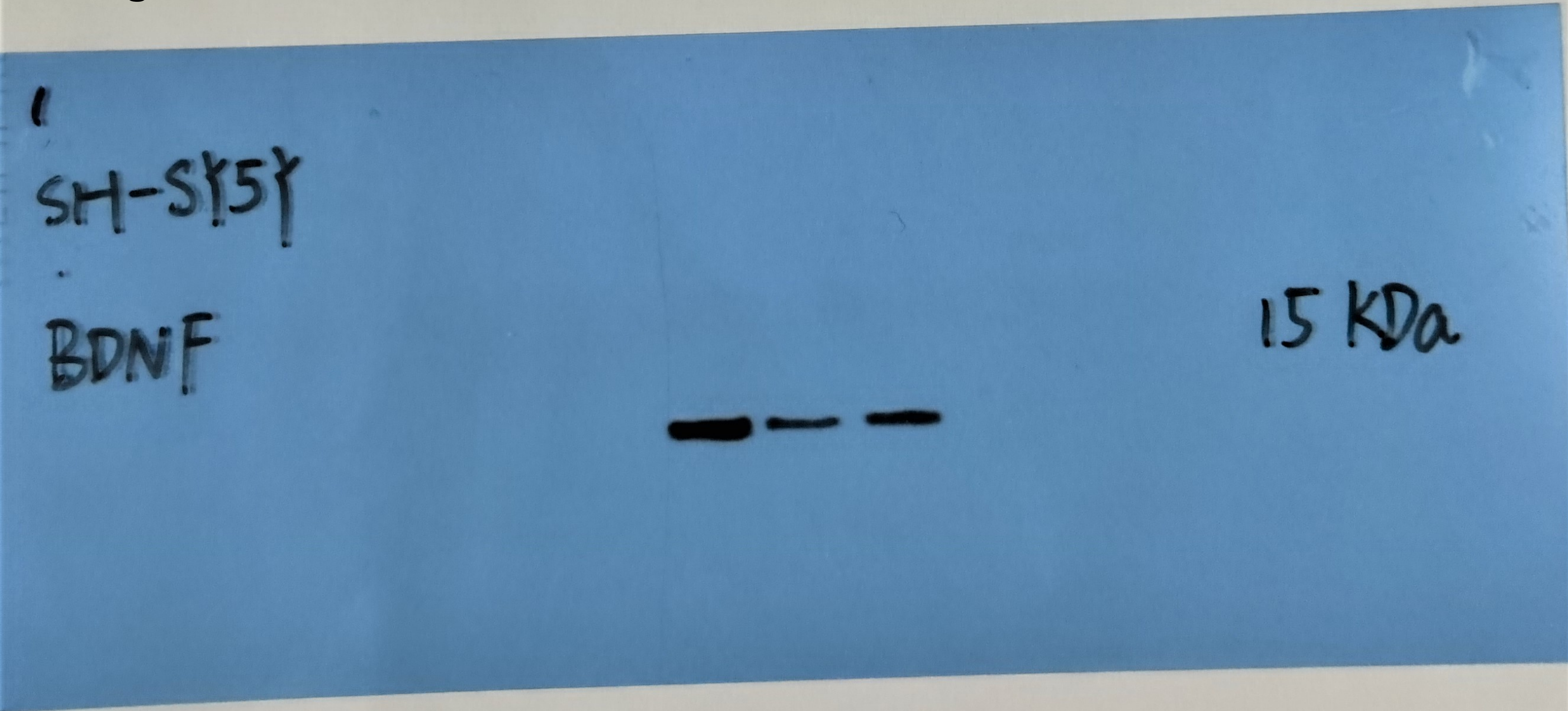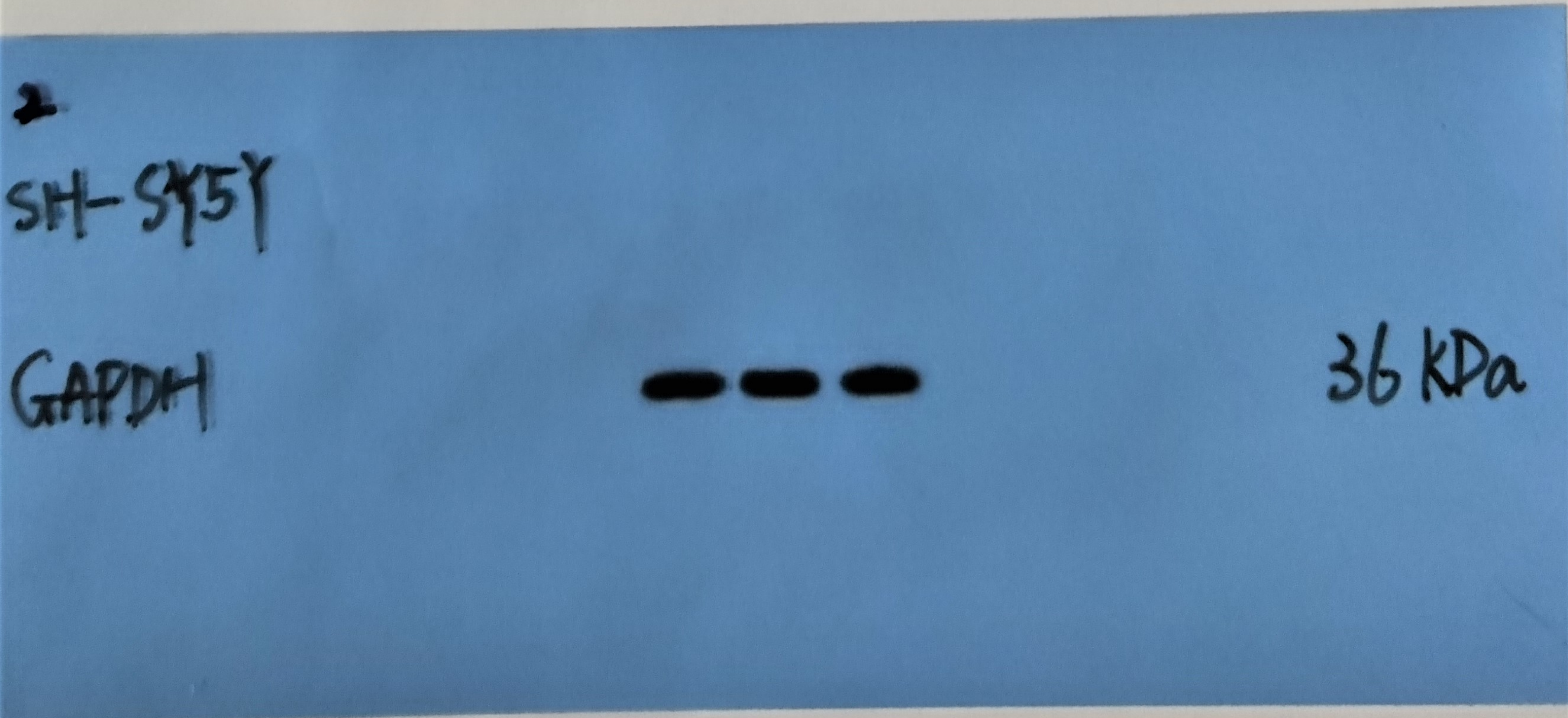

Supplement: Supplementary file 1 — Additional file 1. [file 12871_2022_1810_MOESM1_ESM.zip › WB original data 3.pdf]

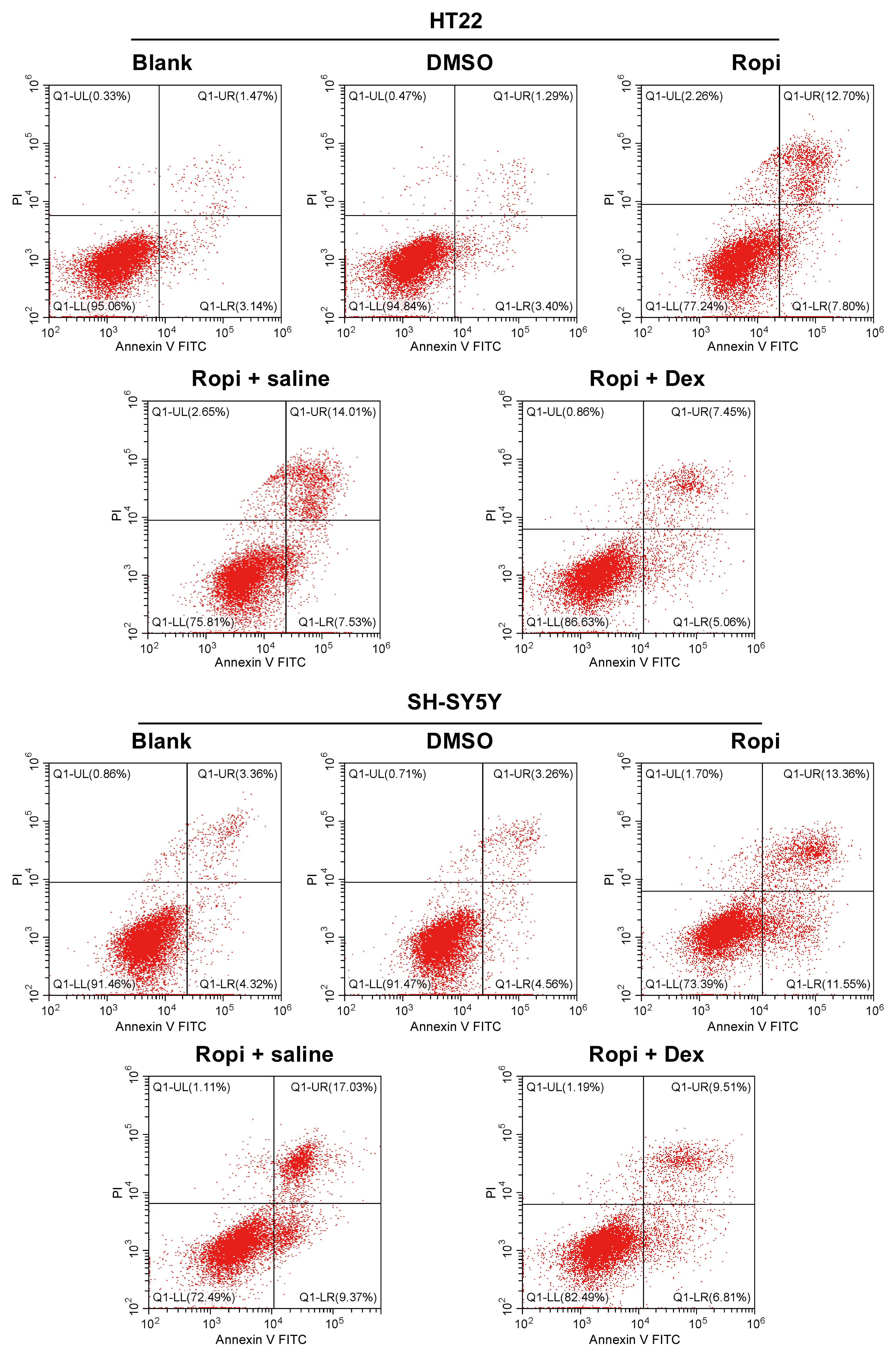

Supplement: Supplementary file 2 — Additional file 2: Supplementary Fig. 1. [file 12871_2022_1810_MOESM2_ESM.tiff]

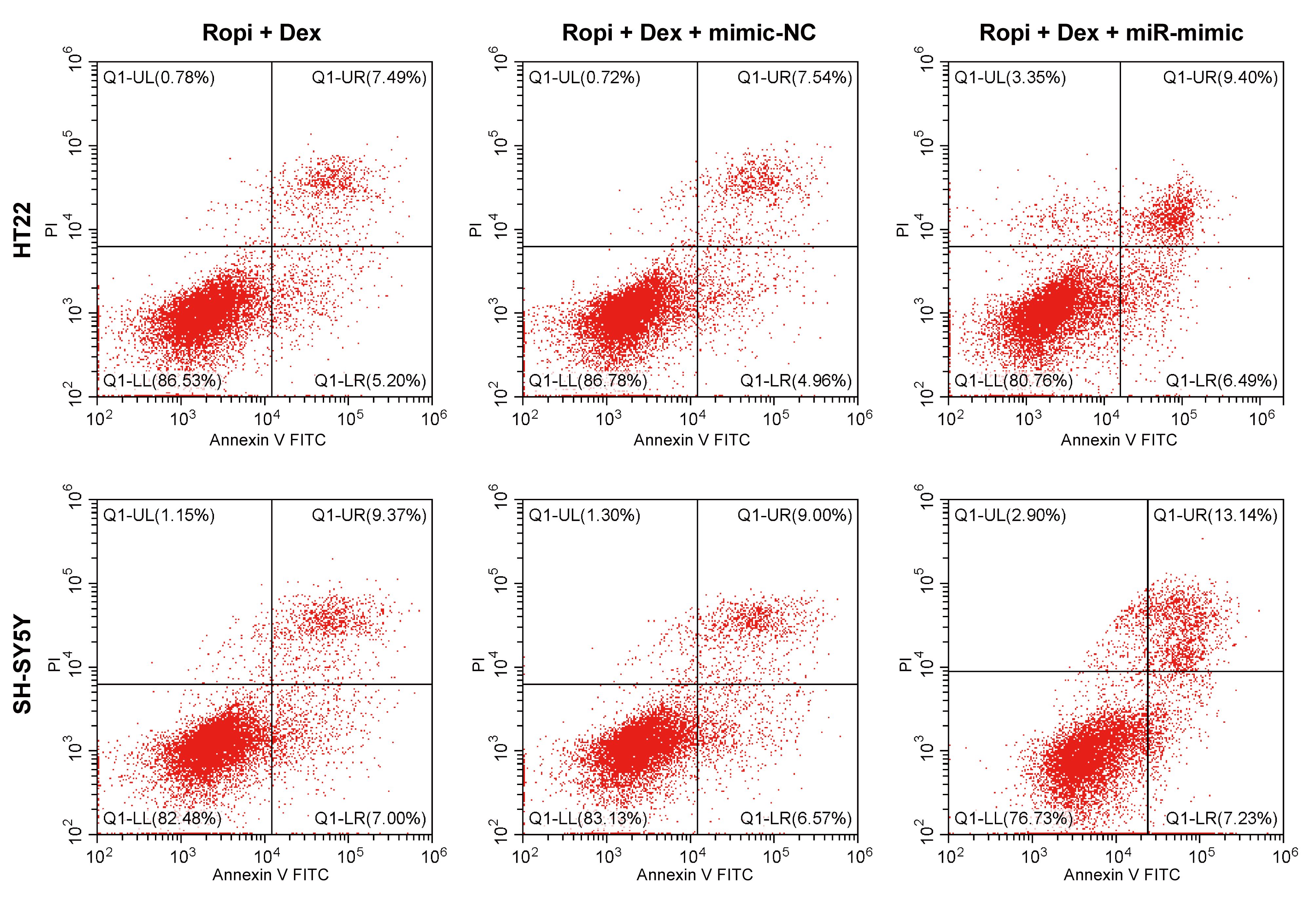

Supplement: Supplementary file 3 — Additional file 3: Supplementary Fig. 2. [file 12871_2022_1810_MOESM3_ESM.tiff]

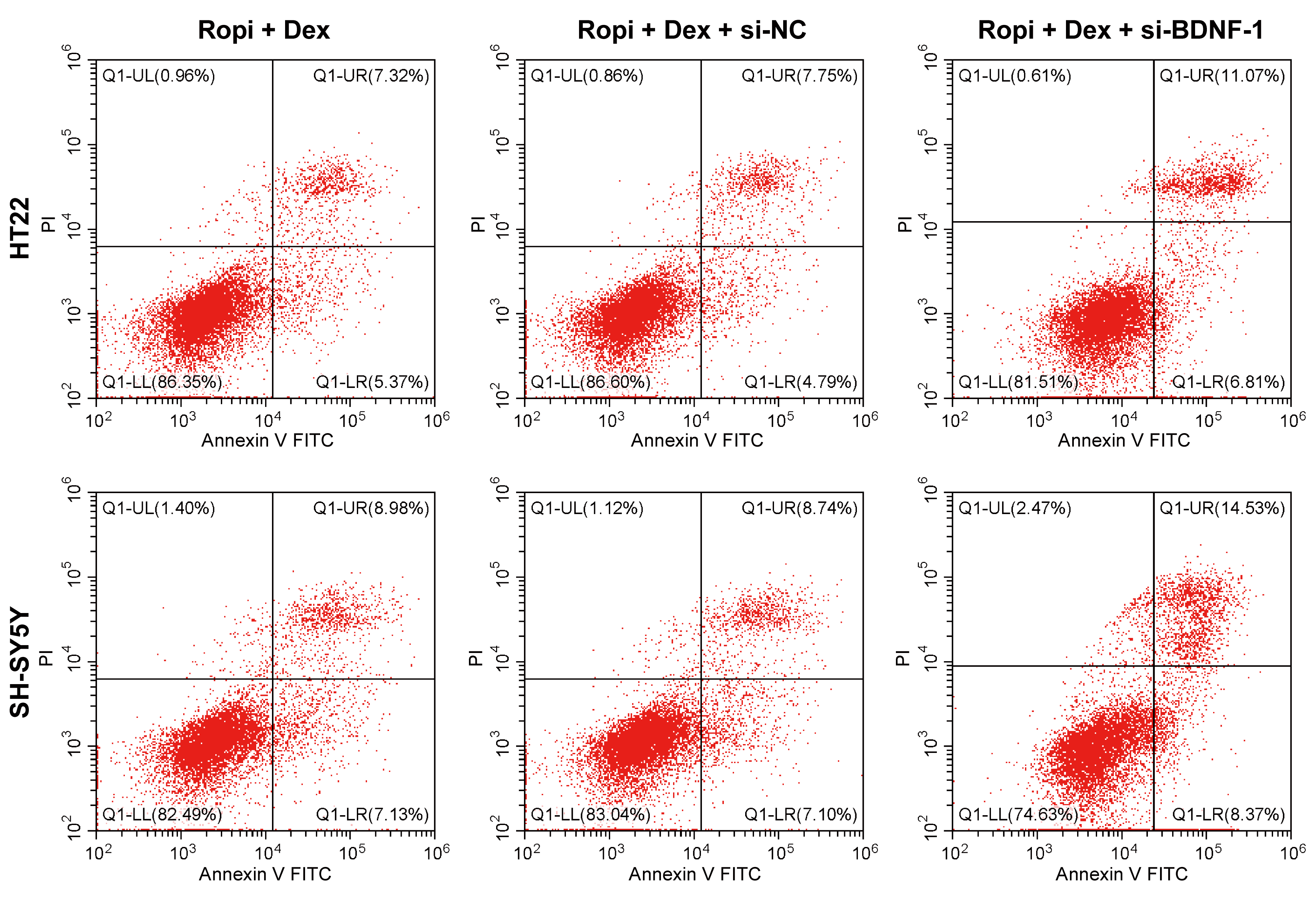

Supplement: Supplementary file 4 — Additional file 4: Supplementary Fig. 3. [file 12871_2022_1810_MOESM4_ESM.tiff]
